# Transcriptional states define dependencies and therapeutic vulnerabilities in head and neck cancer

**DOI:** 10.1038/s41698-026-01509-8

**Published:** 2026-05-25

**Authors:** Joel M. Vaz, Songli Zhu, Mateo Useche, Lauren Shih, Emily Marchiano, Slobodan Beronja, Bruce E. Clurman, Cristina Rodriguez, Brittany Barber, Marina Chan, Taranjit S. Gujral

**Affiliations:** 1 Human Biology Division, Fred Hutchinson Cancer Center, Seattle, WA USA; 2 University of Washington Medical Center, Seattle, WA USA; 3 Clinical Research Division, Fred Hutchinson Cancer Center, Seattle, WA USA; 4 Department of Pharmacology, University of Washington, Seattle, WA USA

**Keywords:** Biomarkers, Cancer, Computational biology and bioinformatics, Oncology

## Abstract

Molecular heterogeneity in head and neck squamous cell carcinoma (HNSCC) is well recognized, yet existing subtype frameworks remain largely descriptive and have not translated into therapeutic decision-making. Here, we establish a mechanistic platform that converts transcriptomic diversity into drug-actionable tumor states. Integrating multi-cohort RNA-seq from 727 tumors across five independent datasets, genome-scale CRISPR dependency maps, and pharmacologic screening, we define distinct tumor survival circuits across HPV-negative HNSCC and nominate subtype-matched therapeutic strategies. These circuits encompass a proliferative axis (MYC, MET/FAK, inflammatory and translational programs), an epithelial-differentiated/adhesion program, an EMT-like state with stromal activation, and mitochondrial/oxidative metabolic states, each mapping to selective liabilities (e.g., mitotic/autophagy control, ERBB/PI3K and cadherin signaling, OXPHOS/mitochondrial translation, and G2/M-integrin-Notch pathways, respectively). We then develop a transcriptomic predictor of EGFR-inhibitor response using machine learning and validate it in prospectively collected, fresh patient-derived 3D microtumors. The resulting 13-gene signature identifies erlotinib-responsive tumors (R = 0.93) and maps biologically to an epithelial-differentiated state, outperforming EGFR expression alone. Our study establishes a subtype-to-dependency-to-therapy framework, enabling precision stratification and providing a clinically feasible path for prospective biomarker deployment.

## Introduction

Head and neck squamous cell carcinoma (HNSCC) remains a major global health burden, with ~600,000 new cases annually and limited progress in survival, particularly among human papillomavirus (HPV)-negative tumors, where five-year survival still hovers near 50% despite multimodal therapy^1^. Over the past decade, large-scale genomic and transcriptomic efforts have revealed substantial biological heterogeneity in HNSCC, identifying discrete molecular programs and refining tumor classification beyond anatomic site^2–7^. These studies have defined canonical subtypes, including classical, basal, mesenchymal, and atypical/HPV-related groups, and provided foundational insights into disease biology. However, these subtype frameworks have largely remained descriptive. They illuminate tumor diversity but do not yet provide a mechanistic basis for therapy selection, nor do they accurately predict clinical response or guide patient stratification.

The critical challenge is therefore not simply to catalogue heterogeneity, but to operationalize it. Despite extensive molecular profiling, HNSCC has lagged behind other solid tumors in realizing precision oncology. Actionable genomic drivers are uncommon; common alterations such as TP53, CDKN2A loss, and NOTCH pathway mutations lack targeted therapies; and current biomarkers, including PD-L1 expression and tumor mutational burden, incompletely predict response to immunotherapy^8,9^. Likewise, although EGFR is highly expressed in HNSCC and EGFR inhibitors have been evaluated clinically, therapeutic benefit remains modest and inconsistent, underscoring the inadequacy of single-marker approaches and the need for biological context.

Here, we explore how HNSCC molecular subtypes relate to functional dependencies and therapy response. We integrate five independent RNA-sequencing cohorts to generate one of the largest unified transcriptomic atlas of HNSCC to date and derive reproducible molecular states across HPV-positive and HPV-negative disease. We then superimpose genome-scale CRISPR gene-essentiality maps and unbiased pharmacologic screening data to uncover survival circuits and subtype-specific therapeutic liabilities. This integrative strategy resolves the well-known basal and classical categories into mechanistically distinct tumor states, distinguished by proliferative signaling, epithelial differentiation, immune-stromal composition, and metabolic wiring, transforming prior descriptive labels into functional tumor identities with clear biological underpinnings and therapeutic implications.

Finally, we operationalize these biological states to create a clinically implementable prediction framework. Leveraging subtype-informed transcriptomic features, we build and rigorously cross-validate a machine-learning model for EGFR-inhibitor response, then prospectively test and validate this model in fresh, patient-derived 3D microtumors. This direct demonstration that molecular predictors trained in silico can forecast therapeutic sensitivity in primary human tumor tissue represents a key conceptual shift, moving beyond descriptive associations toward a practical precision-oncology framework in which tumor state informs biological dependency and, ultimately, treatment response.

Together, this work provides a principled path to translate tumor heterogeneity into therapeutic decisions in HNSCC. More broadly, it offers a generalizable blueprint for converting multi-omic cancer taxonomies into functional states that guide rational drug selection, model-informed clinical trial design, and prospective biomarker deployment across solid tumors where molecular subtypes are known but not yet actionable.

## Results

### Defining molecular tumor states in HNSCC through multi-cohort transcriptomic integration

**Fig. 1:**
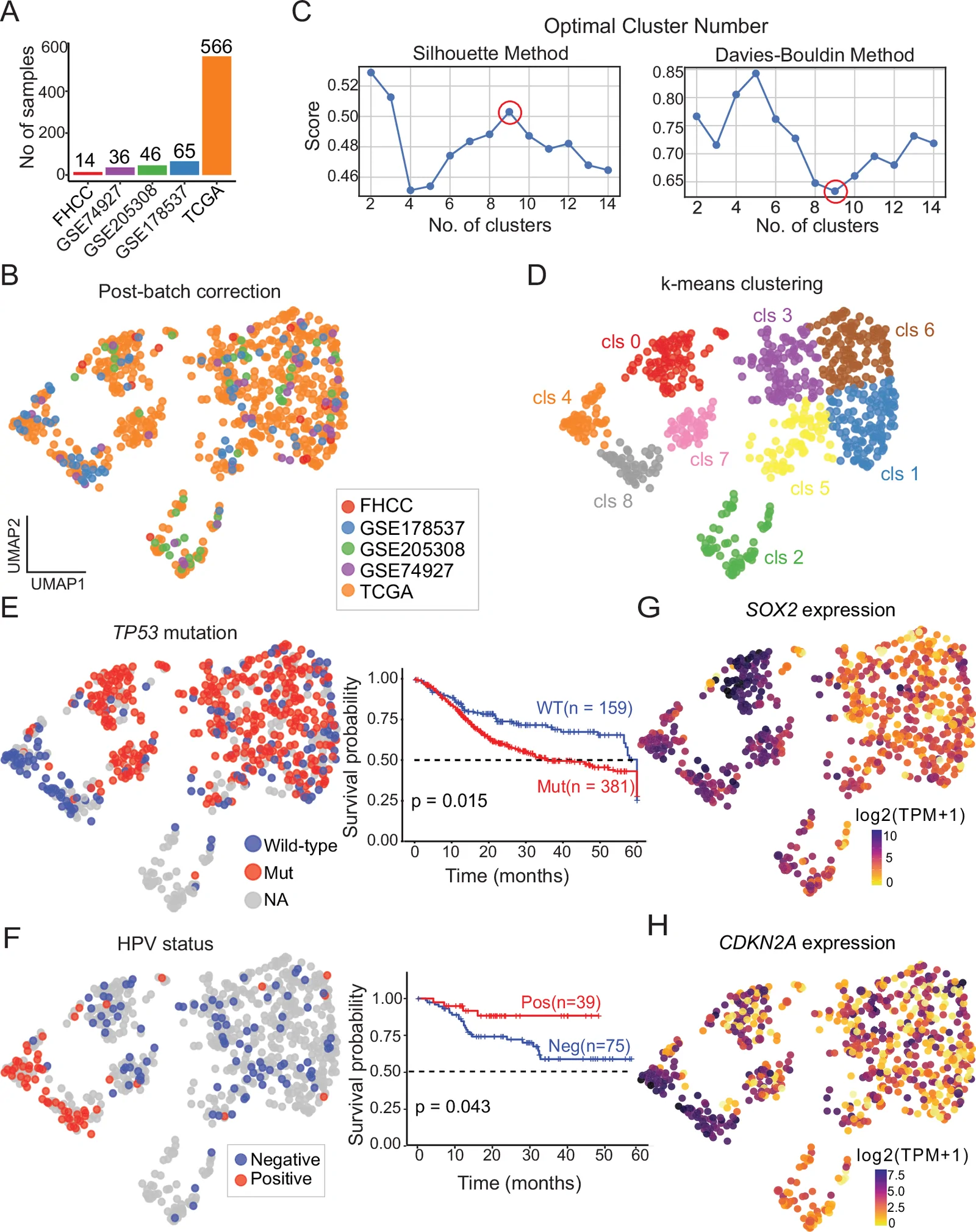
Transcriptomic clustering of HNSCC identifies nine molecular subgroups with distinct genetic and clinical features. **A** Bar plot showing the number of RNA-seq samples collected from different datasets, including Fred Hutch Cancer Center (FHCC), GEO datasets, and TCGA. **B** UMAP visualizing HNSCC samples after batch correction, with colors indicating the dataset of origin. **C** Optimal cluster number determination using the Silhouette (left) and Davies-Bouldin (right) methods. The Silhouette index is maximized, and the Davies-Bouldin index is minimized at k = 9. **D** K-means clustering of the integrated dataset at optimal cluster number, with each color representing a distinct cluster. **E** UMAP showing TP53 mutation status (wild-type vs. mutant), alongside Kaplan-Meier survival curves comparing overall survival between the two groups. **F** UMAP indicating HPV status (negative vs. positive), with the corresponding Kaplan-Meier survival curves for each group. **G** UMAP showing SOX2 log2-TPM expression levels across the samples. **H** UMAP displaying CDKN2A log2-TPM expression levels across the samples.

To comprehensively characterize the molecular landscape of HNSCC, we integrated RNA-seq data from five independent cohorts, FHCC, GSE74927, GSE205308, GSE178537, and TCGA^10^, yielding a total of 753 samples (Fig. 1A, Tables S1 and S2). Raw count data from TCGA were downloaded via the GDC portal, while all other raw files were obtained from the NCBI GEO database. After alignment using STAR^11^, samples with fewer than 60% uniquely mapped reads were excluded, resulting in a final dataset of 727 samples. To account for differences across data sources, we aligned and quantified gene expression (Fig. S1A), then applied batch correction using ComBat-Seq^12^. UMAP visualization after correction showed well-mixed samples from different cohorts, indicating successful harmonization (Fig. 1B). Next, we identified the optimal number of clusters by comparing Silhouette and Davies-Bouldin scores, both of which supported *k* = 9 (Fig. 1C). The Silhouette score measures how well each sample fits within its assigned cluster versus others, with higher values indicating better-defined clusters, while the Davies-Bouldin index evaluates intra-cluster similarity and inter-cluster separation, where lower values suggest more distinct clustering. Unsupervised k-means clustering at *k* = 9 partitioned the dataset into nine distinct subgroups (Fig. 1D). Although we also tested alternative methods including HDBSCAN, Gaussian mixture modeling, and hierarchical clustering (Fig. S1B-S1D), we selected k-means for its ability to define multiple clusters and its strong support from both evaluation metrics.

To assess clustering robustness, we generated more than 450 UMAP embeddings across varying parameter settings (Tables S3). Silhouette analysis consistently supported an optimal cluster number between *k* = 6-9, with *k* = 9 frequently among the highest-scoring solutions (Fig. S1E-S1G). In a half-split validation, UMAP trained on 50% of samples preserved the overall structure when the remaining samples were projected onto the embedding, with samples mapping close to their original cluster regions (Fig. S1H).

To explore clinical and molecular features associated with each cluster, we overlaid key annotations onto the UMAP. *TP53* mutation status, available for ~82% of samples, was enriched across most clusters, except for clusters 4 and 8 (Fig. 1E and Fig. S2A). As previously reported^13^, patients with *TP53* mutations had significantly poorer survival. HPV status, determined by p16 testing in ~17% of samples, revealed that HPV-positive tumors localized to distinct regions in the UMAP, particularly in clusters 4 and 8 where *TP53* mutations were less common (Fig. 1F and Fig. S2B). These tumors, as expected^14,15^, were associated with better survival outcomes. This inverse relationship between HPV positivity and *TP53* mutation was further supported by ISH data (Fig. S2E). Smoking history was more broadly distributed but appeared less common in clusters 4 and 8 (Fig. S2C, S2D). Overlaying tumor anatomical subsites onto the UMAP revealed patterns consistent with prior reports^3,15^, with HPV-positive tumors predominantly originating from the oropharynx and laryngeal tumors largely HPV-negative and enriched in the metabolism-associated subtype (Fig. S2F). However, multiple subsites were distributed across clusters, indicating that the observed subtype structure reflects broader transcriptional programs rather than anatomical origin alone.

The Sex-determining region Y-box 2 (SOX2) gene, a key regulator of embryonic stem cell fate, has been associated with improved prognosis in patients with HNSCC^16^. When SOX2 expression was overlaid onto the UMAP, higher expression was observed in clusters 0, 7, 4, and 8 (Fig. 1G). In contrast, loss or mutation of *CDKN2A*, a common alteration in HPV-negative tumors^17^ was generally associated with lower expression across most clusters, except for clusters 4 and 8 (Fig. 1H), which are enriched for HPV-positive tumors. The mutual exclusivity between HPV positivity and TP53 mutation and/or CDKN2A loss observed in our distinct cluster has been previously described^18^. Cluster 2 predominantly contained normal samples; thus we designated it as the non-tumor cluster. Overall, these findings align with established models of HNSCC pathogenesis, where HPV-positive tumors form distinct molecular subgroups. Although clusters 1 and 6 contained a subset of tumors with unknown HPV status, these samples co-localized with predominantly HPV-negative clusters and showed enrichment of TP53 mutations, a hallmark of HPV-negative HNSCC. In our analysis, we identified two clusters among HPV-positive tumors and six distinct clusters among HPV-negative HNSCC.

**Fig. 2:**
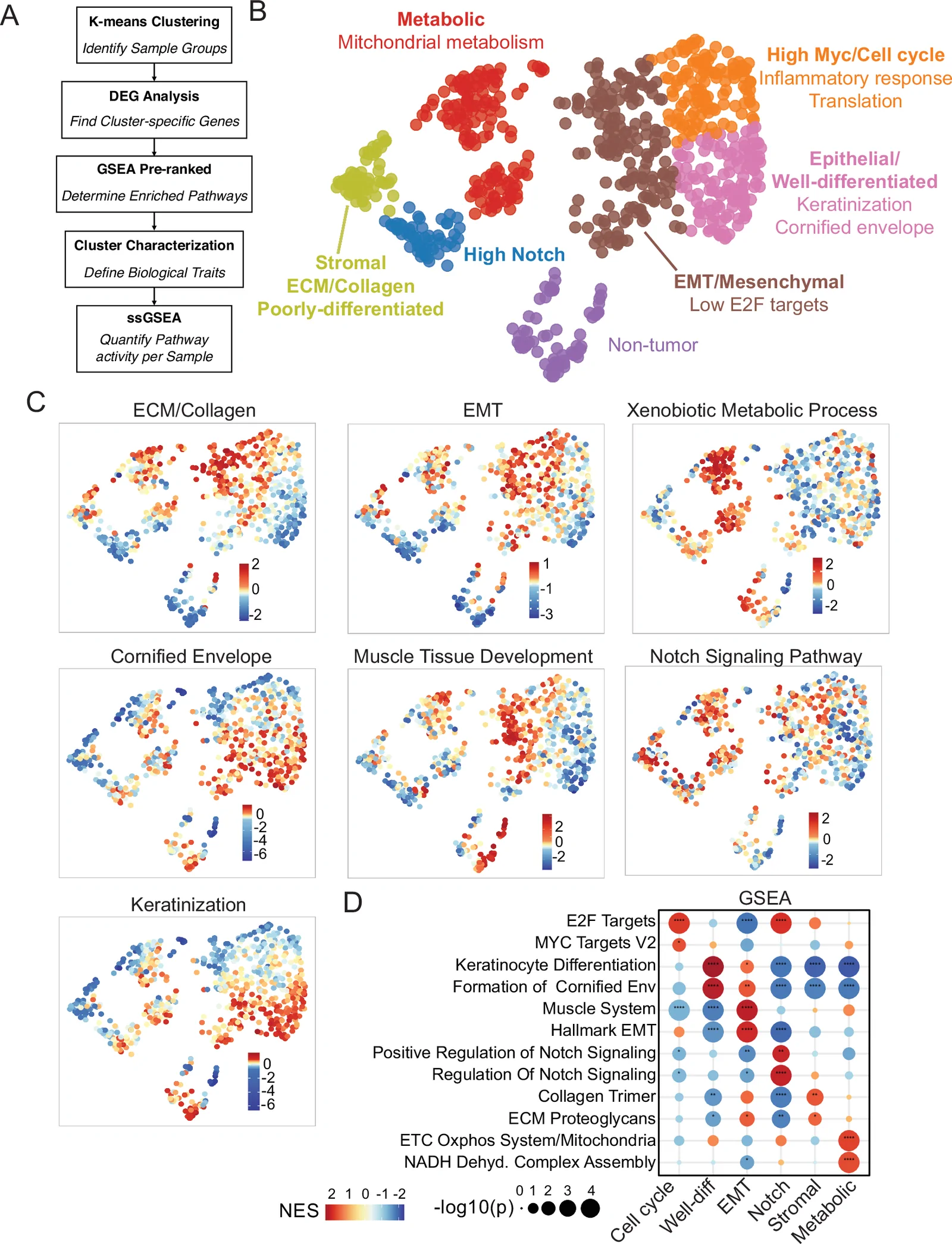
Transcriptomic subtypes of HNSCC reveal distinct biological programs and pathway enrichments. **A** Overview of the workflow. K-means clustering was applied on the integrated RNA-seq dataset to define molecular subgroups. Differential expression analysis was then used to identify cluster-specific genes. Pre-ranked GSEA was applied to determine enriched biological pathways and subtype traits. **B** UMAP visualization of HNSCC samples, color-coded by molecular subtype. Each cluster is annotated with defining biological programs, including metabolic, EMT/mesenchymal, epithelial/well-differentiated, stromal/ECM, and high Notch signaling features. **C** UMAP plots showing single-sample GSEA enrichment scores (z-score normalized) for representative pathways, including ECM/collagen remodeling, EMT, xenobiotic metabolism, cornified envelope, muscle tissue development, Notch signaling, and keratinization. **D** Bubble plot summarizing average pathway enrichment across subtypes (from GSEA Pre-ranked). Bubble size reflects significance (-log10 p-value) and color corresponds to normalized enrichment score (NES). Significance levels are derived from a permutation-based test. ns: p ≥ 0.05, *p < 0.05, **p < 0.01, ***p < 0.001, ****p ≤ 0.0001.

To investigate the biological underpinnings of the HNSCC subgroups identified through unbiased clustering methods, we performed a multi-step transcriptomic analysis (Fig. 2A). First, we conducted differential gene expression (DEG) analysis to identify genes that were significantly up- or downregulated in each tumor cluster compared to all other tumors. Using these DEGs, we generated pre-ranked gene lists for each cluster and applied GSEA-PreRanked^19^ to assess pathway enrichment across multiple gene set collections, including Hallmark, KEGG, Reactome, Biocarta, ImmuneSigDB, and Gene Ontology. Next, we used single-sample Gene Set Enrichment Analysis (ssGSEA) to compute pathway activity scores for each individual tumor sample. These scores were projected into UMAP space to visualize how specific biological programs are distributed across different tumor subtypes (Fig. 2A).

Among the two HPV-positive clusters, both exhibited active Notch signaling; however, they were distinguished by differences in stromal composition. Cluster 0 showed strong Notch pathway activity, enrichment in cornified envelope genes, and low stromal signatures, features consistent with a predominantly epithelial phenotype (Fig. 2B). In contrast, Cluster 8 also displayed Notch signaling but was enriched in stromal-related pathways, including extracellular matrix (ECM) remodeling (Fig. 2B), indicating a more mesenchymal-like or immune-infiltrated microenvironment.

Among the six HPV-negative clusters, we identified distinct biological subtypes based on pathway enrichment patterns (Tables S4). Cluster 6 was marked by strong cell cycle and MYC signaling, along with activation of MET/FAK signaling, inflammatory pathways, and components of the translational machinery; features indicative of a highly proliferative and potentially immune-modulated subtype (Fig. 2C). In contrast, Cluster 1 exhibited signatures of a well-differentiated epithelial phenotype, including enrichment in keratinocyte differentiation, epidermal development, cornified envelope formation, and translation-related pathways (Fig. 2C). Together, Clusters 1 and 6 were grouped into a ‘highly proliferative, well-differentiated module’. Cluster 0 and 7 show strong enrichment in mitochondrial metabolism and oxidative phosphorylation pathways, suggesting a possible dependence on energy production; we refer to this as ‘MT metabolism-enriched module’. While both clusters are linked to metabolic activity, only Cluster 0 showed enrichment of Wnt signaling. (Fig. 2C). Clusters 3 and 5 showed enrichment in mesenchymal and muscle-related processes, such as epithelial-to-mesenchymal transition (EMT), muscle development, and myogenesis. These clusters also exhibited low expression of E2F target genes, consistent with reduced cell cycle activity and increased potential for migration or invasion, leading us to classify them as the ‘EMT module’. Thus, based on pathway enrichment and underlying biological processes, the six HPV-negative clusters were grouped into four distinct modules: a highly proliferative, well-differentiated module; an MT metabolism-enriched module; an EMT module; and an immune-modulated proliferative module.

We next evaluated the influence of non-tumor and HPV-positive samples on the HPV-negative tumor map (Fig. S3A). To assess this, we re-clustered the dataset after removing normal tissues (Fig. S3B), HPV-positive samples (Fig. S3C), and both HPV-positive and normal samples (Fig. S3D). Across these analyses, the HPV-negative tumor clusters remained stable and continued to group together while separating from HPV-positive and normal samples when present.

We next evaluated associations between molecular subtypes and clinical outcomes using TCGA HNSCC data. Kaplan-Meier analysis showed that the Notch and Stromal subtypes were associated with improved overall survival compared with other groups, while pairwise comparisons among HPV-negative subtypes did not reveal significant survival differences (Fig. S4A, S4C). Analysis of recurrence showed no significant association between subtype and recurrence frequency (chi-square test, p = 0.179; Fig. S4B)

### Relationship to prior subtyping frameworks

Our refined HPV-negative tumor states build on and extend the TCGA subtypes^3,7^. Consistent with prior work, we recover basal, mesenchymal, and classical patterns (Fig. S3E). However, our integrated analysis separates the TCGA basal group into two mechanistically distinct states, a proliferative MYC/MET-FAK-high program and a well-differentiated epithelial program and subdivides the classical class into two mitochondrial-metabolism states. The mesenchymal class aligns directly with our EMT-enriched state. Together, these refinements further resolve the transcriptional heterogeneity contained within the basal and classical TCGA subtypes.

### Tumor microenvironment signatures distinguish molecular subtypes

**Fig. 3:**
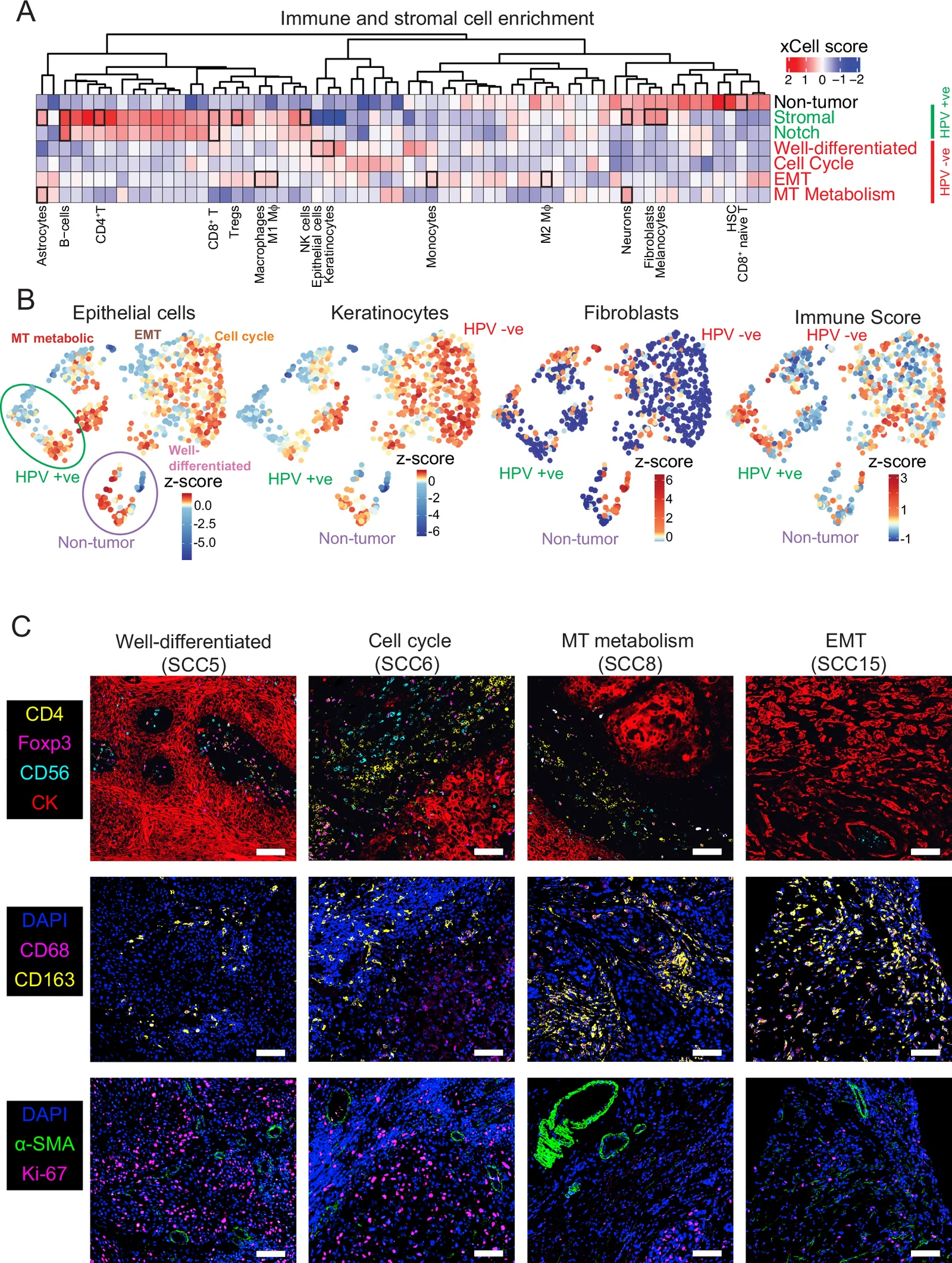
Immune and stromal landscapes distinguish molecular subtypes of HPV-Negative HNSCC. **A** Heatmap of z-normalized xCell scores for 64 immune and stromal cell types across molecular subgroups of HNSCC. Rows correspond to subtypes (non-tumor, MT metabolic, EMT, cell cycle, well-differentiated, Notch, stromal), and columns correspond to inferred cell populations. Red indicates higher enrichment, blue indicates lower enrichment. **B** UMAPs illustrating the predicted enrichment of selected cell types and immune scores at the single-sample level. Each point is colored by the inferred abundance of epithelial cells, keratinocytes, fibroblasts, CD8 + T cells, or overall immune score. **C** Representative multiplex immunofluorescence staining of HPV-negative HNSCC tissues from each major subtype. Panels show epithelial cells (CK⁺), lymphoid cells (CD4⁺, FoxP3⁺, CD56⁺) (top), macrophages (CD68⁺/CD163⁺) (middle), and proliferation and stromal activation (Ki-67⁺, α-SMA⁺) (bottom). Distinct patterns are observed across well-differentiated, cell cycle, mitochondrial metabolism, and EMT subtypes, highlighting differences in epithelial content, immune infiltration, proliferative activity, and stromal composition.

Having identified four distinct modules among HPV-negative tumors and two among HPV-positive tumors, we next sought to characterize the contribution of specific cell types, particularly stromal and immune cells, across these subtypes. We performed cellular deconvolution using xCell^20^, a gene signature-based method that estimates the relative enrichment of 64 immune and stromal cell types in bulk tissue transcriptomes (Table S5). This analysis revealed varying degrees of infiltration by epithelial cells, fibroblasts, macrophages, T cells, and other immune populations across the molecular subgroups (Fig. 3A). As expected, the HPV-positive subtypes showed increased enrichment of immune cells, including CD4+ and CD8 + T cells, consistent with an “immune subtype” classification^21^. Among these, the stromal-enriched HPV-positive cluster showed higher fibroblast scores and lower enrichment of epithelial cells and keratinocytes, suggesting a more mesenchymal-like tumor microenvironment. In contrast, non-tumor samples showed higher enrichment of hematopoietic stem cells (HSCs) and naive CD8 + T cells, consistent with the presence of normal tissue-associated immune populations.

Among the HPV-negative tumors, the well-differentiated and cell cycle-driven modules displayed higher enrichment of epithelial cells and keratinocytes, reflecting a more epithelial-like phenotype (Fig. 3B). In contrast, the EMT module showed elevated levels of M2-like macrophages, the highest among all subtypes, consistent with more immunosuppressive and pro-invasive microenvironment. Interestingly, the mitochondrial metabolism-enriched module exhibited low enrichment of most immune and epithelial cell signatures but showed relatively higher enrichment of astrocyte- and neuron-like signatures. These signals likely reflect neural-like transcriptional programs captured by the deconvolution approach rather than the presence of true neural cell populations.

Next, we sought to evaluate cell type composition and stromal characteristics using multiplex immunofluorescence analysis of freshly collected HPV-negative HNSCC samples. We performed COMET staining with a 20-marker panel to assess epithelial, immune, and stromal features across subtypes (Fig. 3C, Table S6). The well-differentiated subtype exhibited strong cytokeratin (CK) positivity, consistent with an epithelial-rich phenotype. In contrast, the mitochondrial metabolism subtype showed reduced CK signal, reflecting loss of epithelial characteristics. Immune profiling revealed that CD4⁺ T helper cells, FoxP3⁺ regulatory T cells, and CD56⁺ NK cells were reduced in mitochondrial metabolism and EMT-enriched subtypes, indicating diminished lymphoid infiltration. Macrophage markers CD68 and CD163 were most abundant in the well-differentiated subtype, followed by the cell cycle-enriched subtype, and were reduced in the mitochondrial metabolism and EMT-enriched subtypes, suggesting subtype-specific differences in myeloid composition. Ki-67 staining confirmed elevated proliferative activity in the cell cycle-enriched subtype. Given the limited number of samples analyzed (*n* = 1 per subtype), these findings should be interpreted as qualitative and are intended to provide orthogonal support for subtype-associated features observed in the transcriptomic analysis rather than a quantitative comparison.

### Subtype-specific genetic dependencies in HNSCC

**Fig. 4:**
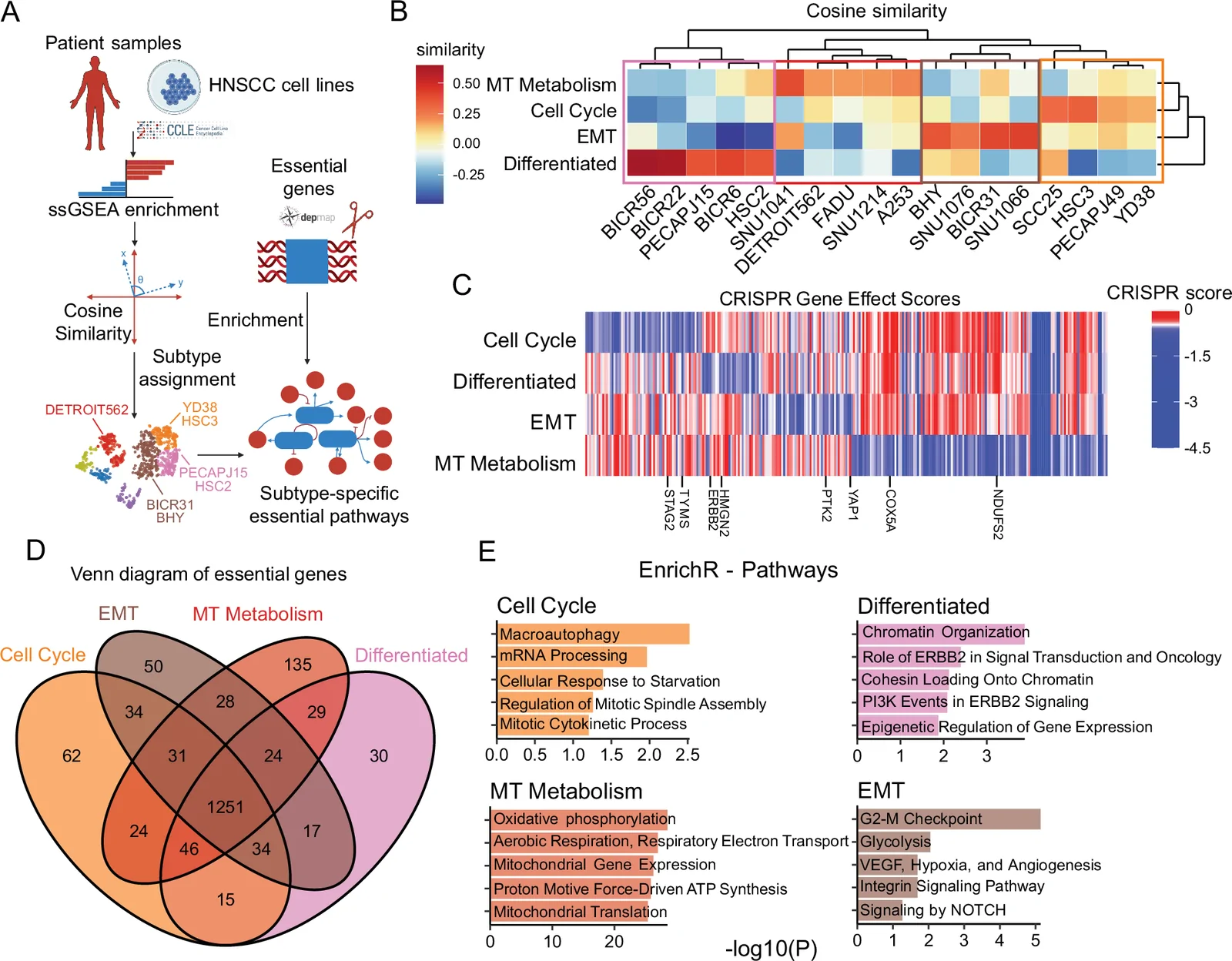
CRISPR Dependency mapping reveals subtype-specific vulnerabilities in HPV-negative HNSCC. **A** Schematic overview of the CRISPR-based analysis used to map cell lines to the pathways essential for their survival. **B** Heatmap of cosine similarity scores between tumor subtype profiles and HNSCC cell lines. Red indicates higher similarity, blue indicates lower similarity, with clustering highlighting correspondence between subtypes and representative lines. **C** Heatmap of CRISPR gene effect scores across subtypes. Red indicates non-essential genes, blue indicates strong essentiality, with subtype-specific dependencies such as STAG2, TYMS, ERBB2, and YAP1 highlighted. **D** Venn diagram of essential gene intersections across HPV-negative subtypes. The largest shared set (n = 1251) reflects core survival genes, while smaller subsets represent subtype-specific vulnerabilities. **E** EnrichR pathway enrichment analysis of subtype-specific essential genes. Distinct pathways were associated with each subtype.

To explore potential therapeutic vulnerabilities associated with HPV-negative HNSCC subtypes, we aimed to identify representative in vitro models with drug response data (Fig. 4A). The Cancer Cell Line Encyclopedia (CCLE) provides a comprehensive resource of molecularly characterized cancer cell lines, including drug sensitivity profiles, making it a valuable tool for identifying subtype-specific dependencies^22^. We began by calculating single-sample GSEA (ssGSEA) scores for 26 available HNSCC cell lines in CCLE using the top 50 upregulated and downregulated pathways previously identified for each cluster. We then computed pairwise cosine similarities between these cell line ssGSEA profiles and those from our integrated tumor dataset (Fig. 4B, Table S7). While many cell lines showed clear maximal similarity to a single subtype, a subset displayed comparable similarity scores across multiple clusters, making confident subtype assignment ambiguous. These ambiguous cases were excluded from subtype-stratified analyses to avoid potential misclassification. The resulting similarity matrix revealed that certain cell lines closely resembled subtypes characterized by cell cycle activity e.g. *SCC25, HSC3, PECAPJ49*, whereas others aligned with metabolic e.g. *SNU1041, DETROIT562, FADU, SNU1214, A253*, or EMT-associated pathways e.g. *BICR56, BICR22, PECAPJ15, BICR6, HSC2*. Based on these profiles, we selected a representative panel of 18 HPV-negative cell lines, ensuring that each subtype was included (Fig. 4B).

To identify subtype-specific dependencies, we utilized CRISPR-based gene effect scores from DepMap, which quantify the degree to which a gene is essential for cell survival. These scores are derived from CRISPR-Cas9 loss-of-function screens, where individual genes are knocked out and the impact on cell viability is measured. A lower gene effect score indicates higher dependency, meaning the gene is critical for the survival of that cell line. A cutoff of z-score < -0.5 is typically used to define essential or genes important for cell growth, distinguishing them from non-essential ones (Fig. 4C). We leveraged this data to identify essential genes for each HNSCC subtype. We defined an essential gene for a given subtype as one that was essential across nearly all representative cell lines, corresponding to at least 75-80% of members (all or all but one) (Table S8). Intersection analysis showed that while some essential genes were shared across subtypes, others were uniquely associated with specific subtypes. For example, *STAG2* and *TYMS* were found to be important in the cell cycle-enriched subtype, *ERBB2* and *HMGN2* in the well-differentiated subtype, *YAP1* and *PTK2* in the EMT subtype, and *COX5A* and *NDUFS2* in the mitochondrial metabolism-enriched subtype.

Next, we examined the overlap of essential genes across HPV-negative HNSCC subtypes using a Venn diagram (Fig. 4D). A large, shared core dominated the landscape (n = 1,251; 84.2% of all essentials), indicating that, beyond subtype-specific features, these tumors converge on a common viability core dependency. Enrichment of this pan-subtype set highlighted fundamental gene-expression and cell-division machinery, including RNA metabolism and processing (mRNA splicing and transport; rRNA processing), ribosome biogenesis and translation (formation and function of 40S/60S subunits; initiation/elongation/termination), DNA replication and repair, and cell-cycle control (G1/S and G2/M transitions, checkpoints, spindle assembly, cytokinesis) (Table S9). Together, these pathways define a housekeeping program centered on transcription, RNA processing, ribosome function, protein synthesis, and orderly cell-cycle progression. While a subset was specific to head and neck disease (*n* = 106), most genes were essential across all cell lines (*n* = 1,145; Fig. S5). We also observed subtype-restricted essentials: mitochondrial-metabolism (*n* = 133), EMT (*n* = 62), cell-cycle (*n* = 50), and differentiated (*n* = 30). Although the total number of CRISPR-defined essentials per cell line was broadly similar across groups, the differentiated subtype showed markedly fewer essentials shared among its lines, suggesting greater functional heterogeneity within this class. These data underscore the coexistence of a common survival core with separable, subtype-specific liabilities that motivate targeted follow-up analyses.

To further investigate these subtype-specific dependencies, we performed pathway enrichment analysis using EnrichR on the uniquely essential genes of each subtype (Fig. 4E). In the cell-cycle subtype, enriched terms included macroautophagy, mRNA processing, cellular responses to starvation, and regulation of mitotic spindle assembly/cytokinesis. Together, these point to a proliferative state that buffers biosynthetic demand and energy stress (autophagy/starvation programs) while remaining dependent on core mitotic machinery. The differentiated subtype was enriched for ERBB2-mediated PI3K signaling, chromatin organization, and cohesin loading, consistent with an epithelial program that maintains lineage identity and cohesion and relies on growth-factor signaling. The mitochondrial-metabolism subtype showed strong enrichment for oxidative phosphorylation, aerobic respiration, mitochondrial gene expression/translation, and proton motive force-driven ATP synthesis, highlighting a mitochondria-centered energy dependence as a key vulnerability. Finally, the EMT subtype was enriched for the G2-M checkpoint, glycolysis, VEGF/hypoxia/angiogenesis, integrin signaling, and NOTCH signaling, indicating a mesenchymal, microenvironment-interactive state that retains checkpoint control, reroutes metabolism, and leverages stromal/angiogenic and adhesion pathways for viability and invasion. Together, these analyses reveal distinct biological processes underlying each subtype and offer potential therapeutic entry points tailored to their specific vulnerabilities.

### Pharmacologic vulnerabilities across molecular tumor states

**Fig. 5:**
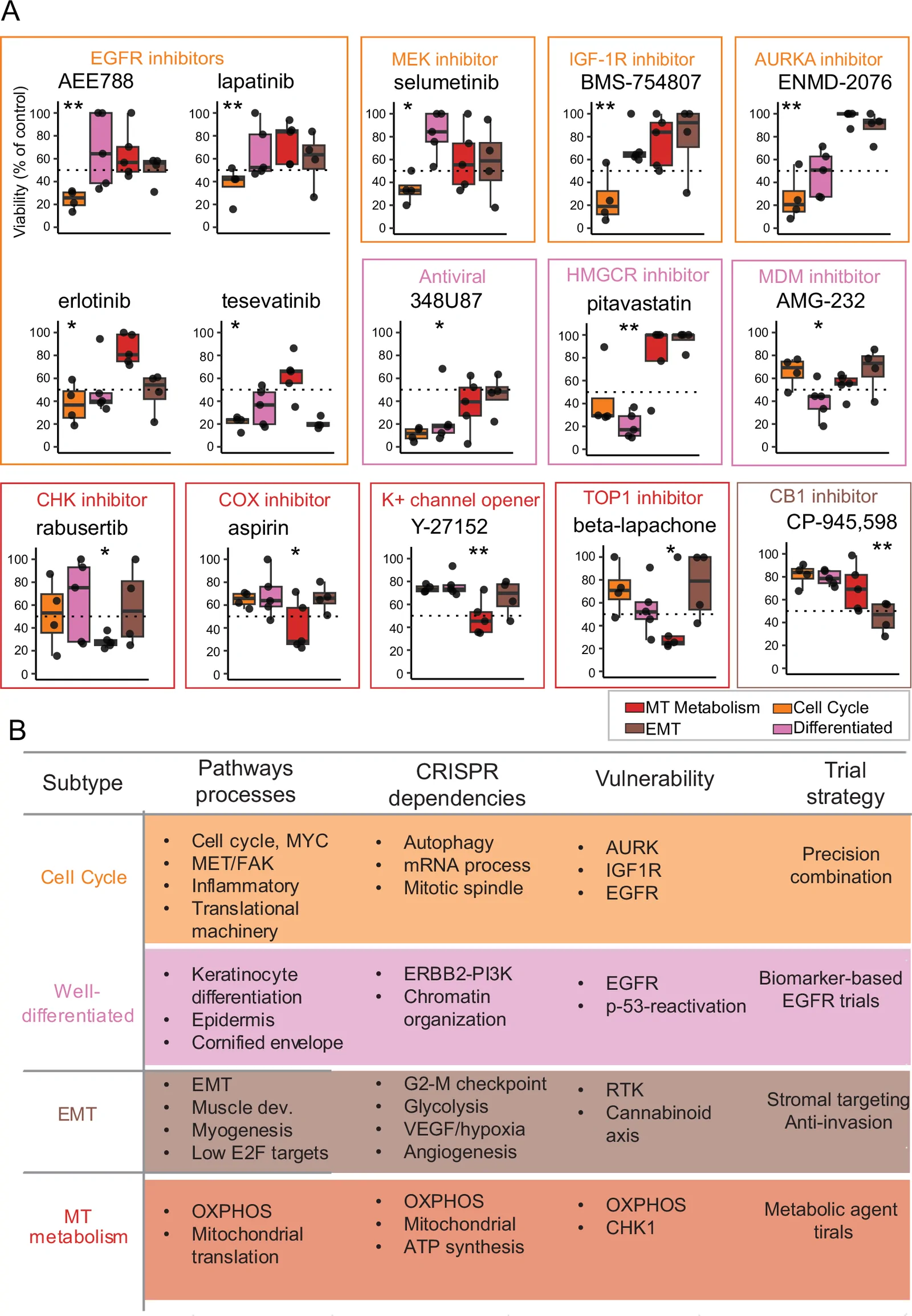
Subtype-specific drug sensitivities reveal distinct therapeutic vulnerabilities in HPV-Negative HNSCC. **A** Boxplots show cell viability after treatment with representative compounds, grouped by subtype assignment. **B** Summary table linking molecular subtypes of HPV-negative HNSCC to their defining biological programs, enriched pathways, candidate therapeutic agents and potential trial strategy.

To investigate pharmacologic sensitivities linked to HPV-negative HNSCC subtypes, we leveraged the PRISM Repurposing drug screen^23^ across representative cell lines and observed subtype-specific response patterns that mirrored the dominant biological pathways of each group (Fig. 5A).

As an example, EGFR inhibitors, including AEE788, lapatinib, and erlotinib exerted potent cytotoxicity in both the Cell Cycle and Differentiated subtype cell lines. This dual efficacy likely reflects and a dependency on upstream growth factor signaling in highly proliferative cell cycle lines (Fig. 4E) and EGFR/ERBB pathway activation in differentiated lines^24,25^. Tesevatinib was the only EGFR inhibitor with notable activity in the EMT subtype.

Consistent with their mitotic dependence (Fig. 4E), cell cycle subtype lines also showed pronounced sensitivity to the MEK inhibitor selumetinib, the IGF-1R inhibitor BMS-754807^26^, and the Aurora A kinase inhibitor ENMD-2076^27^, all of which reduced median cell viability to below 40%. These results highlight the vulnerability of this group to disruption of both mitotic and upstream proliferative signaling. In contrast, several agents exhibited selective efficacy in the well-differentiated subtype cell lines. The antiviral compound 348U87^28^, the HMG-CoA reductase inhibitor pitavastatin, and the MDM2 inhibitor AMG-232^29^ were particularly effective in these cells, reducing median viability to 15–45%. Although sterol metabolism was not among the top transcriptionally enriched pathways in this subtype, cholesterol metabolism is closely linked to keratinocyte differentiation biology, and pharmacologic HMGCR inhibition has been reported to perturb squamous differentiation programs^30^. These sensitivities therefore, may reflect vulnerabilities associated with the differentiated epithelial state rather than direct enrichment of sterol signaling pathways.

The mitochondrial metabolism-enriched subtype cell lines displayed unique sensitivity to compounds that target metabolic stress and redox pathways. Specifically, the CHK1 inhibitor rabusertib, COX inhibitor aspirin, potassium channel opener Y-27152^31^, and the TOP1 inhibitor β-lapachone^32^ each reduced viability below 50% only in this group. These findings are consistent with the subtype’s strong enrichment for oxidative phosphorylation and mitochondrial translation pathways^33^.

Finally, the EMT subtype demonstrated selective vulnerability to the CB1 receptor antagonist CP-945,598^34^, which reduced median viability to ~45%. However, CRISPR knockout of CNR1 did not show a corresponding genetic essentiality in these models, suggesting that this effect is unlikely to reflect an on-target CB1 dependency and may instead arise from off-target activity. Accordingly, we interpret this result as compound-associated sensitivity rather than evidence for a functional role of endocannabinoid signaling in this subtype. Together, these results demonstrate that drug response profiles recapitulate and expand upon the genetic dependencies identified in earlier CRISPR screens. These findings offer a robust preclinical framework for matching HNSCC molecular subtypes to rational, targeted therapeutic strategies (Fig. 5B).

### Predicting EGFR inhibitor response using transcriptomic signatures and patient-derived microtumors

**Fig. 6:**
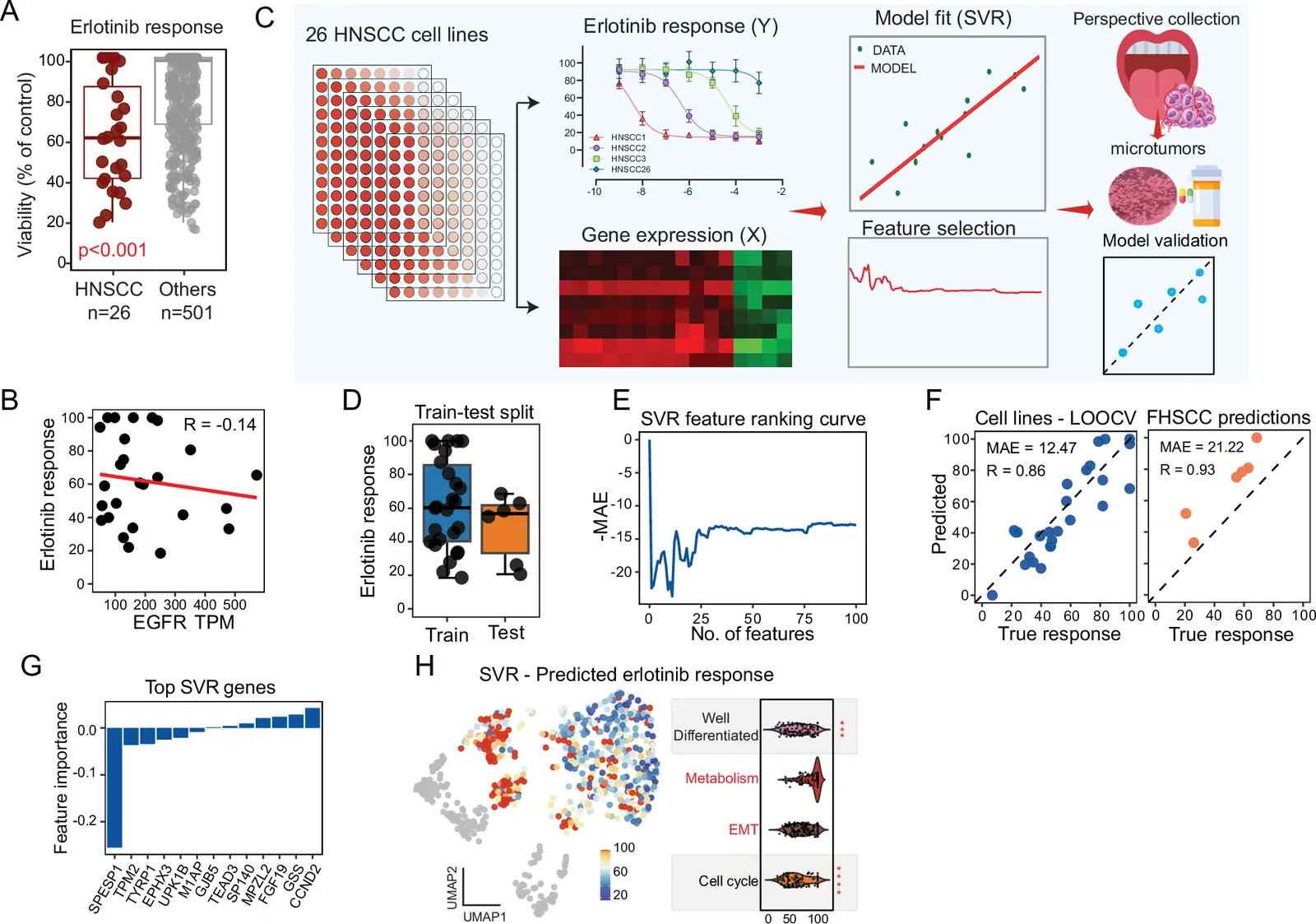
Machine learning identifies gene signatures predictive of EGFR Inhibitor response. **A** Box plot of erlotinib response showing reduced viability in HNSCC lines compared to other cancer cell lines. **B** Scatter plot of erlotinib response versus EGFR mRNA expression (TPM), showing no strong correlation (R = -0.14). **C** Schematic of the supervised learning workflow. RNA-seq from 26 HNSCC CCLE lines was used to train a support vector regression (SVR) model of erlotinib response, which was then applied to predict responses in patient-derived tumors. **D** Box plots of erlotinib response in training (cell lines) and test (patient-derived microtumors) sets, showing reduced viability in clinical samples. **E** SVR feature ranking curve showing cross-validation mean absolute error (MAE) relative to the number of features selected. **F** Scatter plots comparing observed versus predicted responses for cell lines (left, LOOCV; MAE = 12.47, R = 0.88) and patient-derived microtumors (right; MAE = 18.44, R = 0.88). **G** SVR feature importance coefficients highlighting the top predictive genes driving the erlotinib response model. **H** UMAP visualization and violin plots showing SVR-predicted erlotinib viability across HPV-negative HNSCC tumor samples.

Building on the potential drug links in HPV-negative HNSCC subtypes, we next focused on EGFR inhibition, since EGFR is a commonly overexpressed receptor tyrosine kinase in HNSCC and a clinically actionable target (e.g., cetuximab)^35^. EGFR drives downstream MAPK/PI3K pathways that regulate proliferation and survival^36^. However, clinical benefit with EGFR inhibitors is inconsistent, implying that transcriptional context, rather than EGFR levels alone, shapes sensitivity (Fig. 6A). While erlotinib (an EGFR inhibitor) showed stronger responses in HNSCC cell lines compared to all other cancer types (*p* = <0.001), its effects on viability still varied widely within HNSCC, providing a clear rationale to build a model that predicts response from gene-expression features (Fig. 6B).

To identify patients who are more likely to benefit from anti-EGFR therapy, we developed an approach that integrated erlotinib response data from HPV-negative cell lines (training set, *n* = 26), a relatively limited sample size that may constrain model robustness and generalizability, which we addressed through validation in independent patient-derived microtumors, with their baseline transcriptomics profiles (Fig. 6C). We applied a supervised learning framework using support vector regression (SVR) to learn the mapping between gene- expression features and drug response. The best-performing model was then applied to new clinical samples from FHCC cohort (test set, n = 6) to evaluate its accuracy and ability to predict anti-EGFR inhibitor response using transcriptomics data alone (Fig. 6D).

To identify features predictive of erlotinib response, we employed a recursive feature elimination with leave-one-out cross-validation (RFE-LOOCV) framework using SVR, as previously described in Kang et al., 2022^37^. After removing genes with low expression and variance, we subsampled 90% of the training data and performed RFE to rank informative genes. The top 100 ranked genes were evaluated across all cell lines using LOOCV, and the optimal model was achieved using 13 genes, resulting in a minimum cross-validation MAE of 12.47 (Fig. 6E).

The true test of the model is its ability to predict drug response in clinical samples. To this end, we recruited 6 HPV-negative HNSCC cases from the FHCC cohort and evaluated the response to erlotinib using patient-derived 3D microtumors. Microtumors offer important advantages over 2D cell lines because they retain the native tumor microenvironments, preserve multiple cell types and can be prepared immediately from surgical specimens^38^. We have recently shown that drug responses in 3D microtumors outperforms sensitivity testing in 2D cultures^38^. For each case, microtumors were generated and treated with erlotinib at 2.5uM and response was measured using real-time glo, as described previously^39^. In parallel, gene expression profiles from these 6 cases were used to evaluate the in silico prediction accuracy of our model. Our model demonstrated strong generalization to the test set, achieving an MAE of 21.22 and a Pearson correlation (R) of 0.93 in 6 independent clinical samples (Fig. 6F). The final 13-gene predictive signature comprised both sensitivity-associated (*GJB5, TEAD3, SP140, FGF19, GSS, CCND2*) and resistance-associated (*SPESP1, TPM2, TYRP1, EPHX3, UPK1B, M1AP*) features (Fig. 6G). We applied our trained model to predict erlotinib response across HPV-negative tumor samples and observed that the Cell Cycle and Differentiated subtypes were predicted to be significantly more responsive to erlotinib compared with the EMT and Metabolism subtypes (Wilcoxon one-sided rank-sum test) (Fig. 6H). Application of this signature to the integrated tumor transcriptomic landscape, using single-sample enrichment scoring, revealed that the sensitivity-associated gene set was significantly enriched in the well-differentiated subtype (Wilcoxon one-sided rank-sum test, *p* < 0.0001), indicating that elevated expression of these genes may underlie heightened erlotinib responsiveness (Fig. S6A). In contrast, the resistance-associated signature showed preferential enrichment in the mitochondrial metabolism-enriched subtype, suggesting that upregulation of these genes may contribute to the intrinsic erlotinib resistance observed in this subgroup (Fig. S6B).

Several of the selected genes are biologically relevant to cell state regulation. *CCND2*, identified in our top predictive gene set, has been observed to be upregulated in less aggressive, more differentiated squamous epithelial tissues and in contexts of *CCND1* downregulation, suggesting a compensatory role that reflects preserved epithelial differentiation in HNSCC^40^. *TEAD3*, together with *TEAD1*, is essential for maintaining basal keratinocyte proliferation and suppressing terminal differentiation; inhibition of TEAD activity leads to rapid activation of differentiation programs in human epidermis^41^. *MPZL2* mediates cell-cell adhesion in epithelial tissues^42^.

We applied several other machine learning methods to build, train and test models on the same dataset. Random Forest regression achieved a reasonable LOOCV fit (MAE = 20.17, R = 0.69) using only 10 genes (*JPH2, CRIP1, HTR2C, ANOS1, ANXA8, MUC15, KLF4, C11orf71, CPVL, KRT15*) and exhibited strong performance on the clinical test set (MAE = 11.84, R = 0.73; Fig. S6C, S6D). Among the selected features were *KLF4* and *KRT15*, genes linked to differentiation and epithelial identity^43,44^. Using Elastic Net regression, the training fit was modest (MAE = 21.39, R = 0.33 with 25 genes; Fig. S6E) but performance improved on the test set (MAE = 9.33, R = 0.79) (Fig. S6F). Key predictors included *MKI67*, an established proliferation marker and *SCD5*, a stearoyl-CoA desaturase linked to epithelial differentiation^45,46^. XGBoost regression showed moderate performance (CV MAE = 18.21, CV R = 0.35; test MAE = 17.68, test R = 0.42; Fig. S6G, S6H) using a 38-gene model, which included *WNT2B*, a gene implicated in EGFR-WNT signaling cross-talk, particularly in modulating epithelial plasticity and resistance mechanisms^47^.

Together, these results demonstrate that erlotinib sensitivity in HPV-negative HNSCC can be robustly predicted from transcriptomics features. By integrating machine learning with patient-derived 3D microtumors, we developed and validated predictive models that generalize to clinical samples and reveal biologically meaningful gene signatures.

## Discussion

HNSCC remains a biologically diverse and clinically challenging disease in which molecular stratification has not yet delivered therapeutic precision. Here, we developed an integrated approach that connects tumor transcriptomic state to functional dependencies, drug sensitivities, and clinically relevant predictors of targeted therapy response. Our analysis resolves HPV-negative HNSCC into mechanistically distinct tumor states that transcend traditional “basal” and “classical” labels. By harmonizing five independent RNA-seq datasets, we identify subgroups characterized by proliferative signaling (MYC, MET/FAK), epithelial differentiation and adhesion programs, EMT-like features with stromal activation, and mitochondrial metabolic wiring. These states reflect known biological axes in HNSCC yet provide greater resolution and functional interpretability than previous taxonomies. Within this spectrum, a proliferative axis is marked by MYC activity, MET/FAK signaling, inflammatory programs, and translational machinery. MYC is frequently upregulated in HNSCC and linked to aggressive growth, therapeutic resistance, and modulation of the tumor-immune interface^48^, while MET and FAK promote proliferation, invasion, and treatment resistance, and functionally intersect with EGFR signaling to support aggressive disease phenotypes^49^. Co-activation of NF-κB, common in HNSCC, further reinforces survival and inflammatory signaling in this state^50^. In contrast, a second axis reflects a well-differentiated epithelial program, enriched for keratinocyte differentiation, epidermal development, and cornified-envelope genes. Maintenance of epithelial adhesion and cell-cell cohesion, including E-cadherin expression, is associated with more differentiated histology and improved outcomes in HNSCC, providing disease-specific context for this state^51^. These distinct transcriptional identities are not simply descriptive; they correspond to specific viability requirements and therapeutic vulnerabilities when overlaid with genome-scale CRISPR dependencies, underscoring that transcriptional state is tightly linked to dependency architecture and implying different therapeutic entry points within HPV-negative tumors.

Extending these observations, integrating CRISPR essentiality data with these transcriptional states demonstrates that subtype identity corresponds to distinct survival circuitry. While HPV-negative tumors share a core set of viability genes involved in RNA processing, ribosome function, DNA replication/repair, and cell-cycle surveillance^52^, each subtype exhibits selective dependencies. The proliferative state relies on autophagy and mitotic machinery, consistent with prior evidence that autophagy supports HNSCC growth, and that AURKA-driven spindle control contributes to poor outcomes^53^. The well-differentiated epithelial state depends on ERBB/PI3K signaling and cadherin-mediated adhesion, reflecting preserved epithelial programs. The mitochondrial-metabolic state shows reliance on oxidative phosphorylation and mitochondrial translation, in line with reports that OXPHOS sustains survival and therapy tolerance in HNSCC^54^. The EMT-enriched state exhibits dependencies in G2/M regulation, glycolysis, angiogenic signaling, integrin pathways, and Notch activity, consistent with invasive and stromal-interactive biology^55,56^. These findings indicate that transcriptional state maps to discrete functional liabilities, converting subtype distinctions into actionable biological dependencies. As these analyses are based on cell line models, the inferred dependencies reflect tumor cell–intrinsic programs rather than the full complexity of the tumor microenvironment.

A central feature of this study is the translation of computational subtype biology into a clinically deployable tool. We developed and experimentally validated a transcriptomic predictor of EGFR inhibitor response using patient-derived 3D microtumors, a platform that retains native architecture and microenvironmental context. Although the predictive models were trained on cell line data, which do not fully capture tumor complexity, their ability to generalize to primary tumor tissue demonstrates the feasibility of this approach for precision oncology in HNSCC. While EGFR expression alone has historically been insufficient to guide therapy in this disease^57^, our model leverages broader transcriptional programs, particularly epithelial differentiation, to identify tumors likely to respond to EGFR blockade, supporting the development of more effective biomarker strategies. Importantly, while transcriptomic data can be used directly to predict drug response, the subtype framework provides a complementary layer by linking predictive features to coherent biological programs and underlying dependency architecture. This enables interpretability and extends beyond single-drug predictions, as subtype-associated vulnerabilities can inform multiple therapeutic strategies and support biologically meaningful patient stratification. Together, these approaches demonstrate how subtype structure can complement, rather than replace, direct predictive modeling.

Taken together, this work converts transcriptomic heterogeneity in HNSCC into an actionable map that links tumor states to what they depend on and how they respond to drugs and then turns that map into a predictor in patient tissue. Many solid tumors have recognized transcriptomic classes but lack actionable frameworks; our approach offers a template for converting tumor heterogeneity into treatment hypotheses that can be tested in ex vivo systems and, ultimately, in clinical trials. Notably, squamous cell carcinomas from multiple anatomical sites share conserved lineage transcriptional programs, suggesting that some of the functional states identified here may reflect broader squamous epithelial configurations rather than HNSCC-specific phenomena^58^. Further, while our analyses focus primarily on HPV-negative HNSCC, HPV-positive tumors represent a biologically distinct class driven by viral oncogenes and characterized by distinct immune and stromal features. Although some higher-level transcriptional programs may extend across HPV status, the specific dependencies and therapeutic vulnerabilities of HPV-positive tumors are likely governed by different regulatory mechanisms and warrant dedicated investigation. Finally, the distinct histomorphologic patterns associated with these transcriptional states also raise the possibility that subtype identity could be inferred from routine histopathology using AI-based image analysis^59,60^. Overall, the framework presented in this study is immediately extensible to other cancer types where descriptive molecular subgroups exist but are not yet clinically actionable, offering a systematic route to realizing the promise of precision oncology in biologically heterogeneous tumors.

## Methods

### Study participant details

The study was conducted in accordance with the Declaration of Helsinki. Human HNSCC tumor samples were obtained under IRB-approved protocols (00001852 [STUDY00001852]). Tissues were collected with informed consent and de-identified prior to use (Table S10).

### RNA extraction and library preparation

Total RNA was extracted from human tissue using the AllPrep DNA/RNA Mini Kit (Qiagen, 80204) per the manufacturer’s protocol. Fresh or snap-frozen samples were homogenized in RLT buffer with ceramic beads (Omni International, Cat. No. 19-646-3) using the Bead Ruptor 12 at 3-4 M/s for 5 s (1-2 cycles). Following a centrifugation, the resulting supernatant was processed through the kit’s spin columns to sequentially purify genomic DNA and total RNA. RNA was eluted in RNase-free water, and RNA integrity was assessed using the Agilent TapeStation. Samples were stored at −80 °C. Library preparation, poly(A) enrichment, and paired-end (150 bp) sequencing were performed by Novogene on an Illumina HiSeq 2500.

### RNA sequencing data collection

RNA sequencing data for HNSCC was obtained from four different publicly available datasets. Raw reads were obtained from three GEO datasets - GSE74927, GSE205308, GSE178537. The raw RNAseq counts for TCGA data was downloaded from the GDC Portal via UCSC Xena Browser (https://xenabrowser.net/).

### RNAseq pre-processing

Raw SRA reads were first converted to FASTQ format using SRA-Toolkit. Quality control was performed on a total of 187 FASTQ files using FastQC (https://www.bioinformatics.babraham.ac.uk/projects/fastqc/) and MultiQC (https://github.com/MultiQC/MultiQC). Adapter trimming was applied to files as needed using Trimmomatic^61^. Using STAR^11^ and Samtools^62^, reads were aligned to the GRCh38.p13 genome and indexed. We filtered out any samples with fewer than 60% uniquely mapped reads. Raw counts were generated using featureCounts, a component of the Subread tool^63^. Batch correction was performed using ComBat-Seq^12^. Finally, we filtered the dataset to retain only protein-coding genes, using the GENCODE v40 annotation.

### Clustering

For our UMAP analysis, we transformed raw counts into variance-stabilizing transformed (VST) data using the ‘DESeq2’ package^64^. We then filtered the protein-coding genes to remove those with low variance ( ≤ 1) and low expression ( ≤ 1 in more than 20% of samples). The UMAP was constructed using ~3,000 genes and 727 samples.

We then performed principal component analysis (PCA) using the ‘scikit-learn’ package, converting the count data into principal components (PCs). The top 20 PCs, accounting for 100% of the explained variance, were retained for further analysis. For UMAP construction, we used the ‘Manhattan’ distance metric, 16 nearest k-neighbors, a minimum distance of 0.11, and a spread of 5 (random seed 42).

To identify clusters, we applied four different clustering methods: HDBSCAN, Gaussian Mixture Models (GMM), Hierarchical Clustering, and k-means clustering. HDBSCAN was performed using the HDBSCAN tool with the Euclidean distance metric, a minimum of 4 samples, and a minimum cluster size of 20. The optimal number of clusters (k) was determined using four methods: Elbow Method, Silhouette Method, Davies-Bouldin Index (DBI), and Calinski-Harabasz Index (CHI). The optimal cluster number was then applied to all clustering methods. All clustering analyses, except for DBSCAN, were conducted using scikit-learn.

UMAP stability was assessed across >450 runs with varying parameters (n_neighbors, min_dist, and distance metric), with cluster robustness quantified by silhouette indices. Split-half validation was performed by training the embedding on 50% of the samples and projecting held-out samples to evaluate structural preservation.

### Differential expression analysis

Differential expression analysis was performed using the ‘edgeR’ package^65^. Non-tumor samples were excluded from all group comparisons. Gene counts were first filtered using the ‘filterByExpr’ function before normalizing for library size and performing comparisons using the Exact Binomial Negative Test. Differentially expressed genes (DEGs) were defined as those with an absolute fold change > 1.5, FDR < 0.05, and minimum average CPM of 1.

### Enrichment analysis

Enrichment analysis was performed using GSEA-Preranked with ranks defined as (sign) fold change × ( − log10(FDR)) and 1,000 permutations to compute Normalized Enrichment Scores. For subtype characterization, pathways were ranked by NES, and the top 50 up- and downregulated pathways per cluster (FDR < 25%) were retained. Clustering analysis identified 9 transcriptionally distinct groups. Initial GSEA analyses were performed for all clusters separately; however, two clusters exhibited highly overlapping enrichment profiles and were subsequently combined for presentation and downstream pathway analysis to reduce redundancy. The underlying clustering structure remained unchanged.

ssGSEA 2.0 was applied to shortlisted gene sets using Transcripts Per Kilobase Million (TPM) values derived from GENCODE v40 gene lengths. CRISPR gene enrichment was analyzed with EnrichR in R using KEGG, Biocarta, Reactome, Hallmark, WikiPathways, and Gene Ontology (GO) databases^66^.

### Survival analysis

Kaplan-Meier survival analysis was performed to evaluate overall survival differences across HNSCC subtypes. Overall survival data was used for the analysis, with non-tumor samples excluded. To ensure consistency in comparisons, survival time was truncated at 60 months. Patients who died beyond 60 months were censored and assigned a status of “living”. Kaplan-Meier survival curves were generated using the “survival” R package, and statistical significance was assessed using the log-rank test.

### Cell deconvolution

For deconvolution, we used the xCell^67^ webtool and the default 64 immune and stromal subtype gene signatures.

### Cell line projection onto tumor-derived transcriptional states

Because molecular subtypes were derived from bulk tumor RNA-seq data and include tumor microenvironment contributions, cell lines were not re-clustered within the bulk tumor subtype framework, as they do not recapitulate the full tumor transcriptional landscape. Instead, cell line transcriptomes were projected onto tumor-derived, tumor-intrinsic subtype signatures using ssGSEA-based scoring. Similarity between cell line and tumor ssGSEA profiles was quantified using cosine similarity, defined as

$$\cos \left(\theta \right)=\frac{{\sum }_{i=1}^{n}{x}_{i}{y}_{i}}{\left(\sqrt{{\sum }_{i=1}^{n}{x}_{i}^{2}}\right)\left(\sqrt{{\sum }_{i=1}^{n}{y}_{i}^{2}}\right)}$$

where ${x}_{i}$ and ${y}_{i}$ represent pathway-level ssGSEA scores for a given cell line and tumor sample, respectively. This projection-based strategy is conceptually related to prior approaches that align tumor and cell line transcriptomes to enable comparison of intrinsic cancer cell states despite systematic differences between them (e.g., Celligner^68^). Cell lines with ambiguous subtype correspondence were excluded from subtype-stratified analyses.

### CRISPR gene effect scores

We collected the z-normalized gene effect scores for cell lines of our interest from DepMap (https://depmap.org/portal/).

### Drug response modeling

Gene expression (log₂(TPM + 1)) from CCLE HNSC cell lines was filtered for genes with mean ≥ 0.5, variance ≥ 1, expression ≥ 1 in ≥ 5 samples, and variance-over-mean ≥ 0.1. These features were paired with experimentally derived erlotinib response values (0-100% viability; 100 = no effect). Although larger numbers of HNSCC cell lines are available in DepMap, the PRISM drug response dataset contains substantial missing values across both cell lines and perturbations. To ensure a consistent dataset for downstream analyses, we constructed a complete-case matrix by removing cell lines and drugs with excessive missing values. This filtering produced a dataset without missing entries across all perturbations, resulting in 26 HNSCC cell lines and 5,396 perturbations used for modeling. CCLE and clinical tumor samples (n = 33) were batch-corrected with ComBat-seq. Models were exclusively trained on CCLE data and evaluated on patient samples.

Hyperparameters for Random Forest and XGBoost were optimized with Optuna v3.0.3^69^. Recursive feature elimination (RFE) was used to identify the optimal number of predictors. Model performance was evaluated using mean absolute error (MAE) and the Pearson correlation coefficient (R).

#### SVM

SVR models were trained on standardized data using the RobustScaler and a linear kernel with C = 1.

#### Elastic Net

Same as SVR, with regularization strength (α) = 0.01 and L1 ratio = 0.8.

#### XGBoost

No scaling beyond log₂(TPM + 1); parameters tuned: estimators [10–500], depth [2–10], learning rate [0.01–0.3], subsample [0.6–1.0], colsample_bytree [0.4–1.0]

#### Random Forest

Scaled using a QuantileTransformer prior to model fitting. Hyperparameters were optimized with MAE as the objective function. Parameters tuned: number of estimators [10, 200], maximum tree depth [2,20], and maximum features considered at each split [‘sqrt’, ‘log2’, 0.1, 0.3, 0.5, 0.7].

### 3D microtumors preparation and drug screening

Microtumors were prepared from HNSCC surgical resections as described previously^70^. Briefly, tumor slices were prepared using a Leica VT1200S vibratome (Leica Biosystems, Wetzlar, Germany). To create microtumors, slices (400 μm thickness, 6 mm in diameter) were arranged on a plastic disc and sectioned using a McIlwain Tissue Chopper (Ted Pella, Redding, CA, USA). 3D microtumors were cultured in Williams’ Medium E containing 12 mM nicotinamide, 150 nM ascorbic acid, 2.25 mg/mL sodium bicarbonate, 20 mM HEPES, 50 mg/mL glucose, 1 mM sodium pyruvate, 2 mM L-glutamine, 1% (v/v) ITS, 20 ng/mL EGF, 40 IU/mL penicillin and 40 μg/mL streptomycin. 3D microtumors were treated with Realtime-Glo MT cell viability reagent (Promega, Madison, WI, USA) in 96-well plates. Following a 24-h incubation, luminescence signals were measured using a Biotek Synergy H1 microplate reader (Agilent Technologies, Santa Clara, CA, USA). 3D microtumors were maintained, and real-time viability was measured for 3 to 7 days post-treatment. The viability was determined by calculating the ratio of the signal at the end of the study to the signals from the same tissue before treatment, which was then normalized to the value of the vehicle control 3D microtumors set to 100%.

### Data Visualization

All the visualization for the manuscript was done on R packages ggplot2, pheatmap and python’s matplotlib and Seaborn.

### COMET assay and image analysis

Formalin-fixed, paraffin-embedded tissues were sectioned at 4 microns and mounted onto standard glass histology slides. Dewaxing and antigen retrieval was completed on a Lunaphore COMET PT module. Imaging chips were affixed, and slides were stained and imaged on a Lunaphore COMET following standard procedures^71^. Ten cycles of staining and imaging were completed with 2 antibodies per cycle (Table S11). Following completion of staining/imaging and autofluorescence subtraction, images were analyzed in Visiopharm software. Cell segmentation and phenotype identification was produced using the Phenoplex Guided Workflow. The following phenotypes were identified for analysis: CD4 + CD68- CD163-; FoxP3 + ; Ki-67 + ; CD56 + ; CD68+ or CD163 + ; CK + ; α-SMA + .

### Ethics declarations

Human HNSCC tumor samples were obtained from the Fred Hutchinson Cancer Center cohort under institutional review board (IRB) approval (IRB, Approval No. FHIRB0020169). All samples were fully de-identified prior to use, and the study was determined by the IRB not to constitute human subjects research. Accordingly, the requirement for informed consent was waived.

## Materials availability

This study did not generate new unique reagents.

## Supplementary information

Supplementary information

S1.

S2.

S3.

S4.

S5.

S6.

S7.

S8.

S9.

S10.

S11.

## Data Availability

Raw RNA-seq data have been deposited in the Gene Expression Omnibus (GEO) under accession GSE311507. Preprocessed RNA-seq count data have been provided in Supplementary data (Table S2). Code used to reproduce the clustering analysis (Fig. 1) is publicly available at: https://github.com/gujrallab/HNSCC-clustering. Any additional information required to reanalyze the data reported in this work paper is available from the lead contact upon request.
