# Supplementary material for: Transcriptional states define dependencies and therapeutic vulnerabilities in head and neck cancer
Source: NPJ Precis Oncol. 2026 May 25;10:334. doi: 10.1038/s41698-026-01509-8 (PMC13518800; doi:10.1038/s41698-026-01509-8)
Supplement: Supplementary file 1 [file 41698_2026_1509_MOESM1_ESM.pdf]

Supplementary Information

Transcriptional states define functional dependencies and therapeutic vulnerabilities in head and neck cancer

Supplementary Tables

- **Table S1.** Accessions and sample counts for all datasets, Related to Figure 1.
- **Table S2.** Raw counts of samples in the FH cohort, Related to Figures 1-6.
- **Table S3.** Randomized UMAP parameter settings and corresponding optimal cluster number (k), Related to Figure 1.
- **Table S4.** Top 50 upregulated and downregulated GSEA pathways in each molecular subtype, Related to Figure 2.
- **Table S5.** xCell enrichment scores for all samples in the HNSCC tumor landscape, Related to Figure 3.
- **Table S6.** Quantification of cell populations across HNSCC subtypes, Related to Figure 3.
- **Table S7.** ssGSEA-based cosine similarity scores between landscape subtypes and cell lines, Related to Figure 4.
- **Table S8.** List of genes that are essential in all or all but one members of the subtype, Related to Figure 4.
- **Table S9.** EnrichR results of essential genes, Related to Figure 4.
- **Table S10.** Clinical metadata for study patients, Related to Figures 1-6.
- **Table S11.** COMET antibody information, Related to Figure 3.

Supplementary Figures

- **Fig. S1, related to Fig. 1.** Comparative clustering and evaluation of cluster robustness in HNSCC.
- **Fig. S2, related to Fig. 1.** Clinical and molecular characteristics of HNSCC clusters.
- **Fig. S3, related to Fig. 2.** UMAP structure of integrated HNSCC cohorts and comparison with TCGA classifications
- **Fig. S4, related to Fig. 2.** Clinical outcomes associated with HNSCC molecular subtypes
- **Fig. S5, related to Fig. 4.** Overlap of CRISPR-defined essential genes in HNSCC vs. other cancers.
- **Fig. S6, related to Fig. 6.** Comparative performance of machine learning models for predicting erlotinib sensitivity.

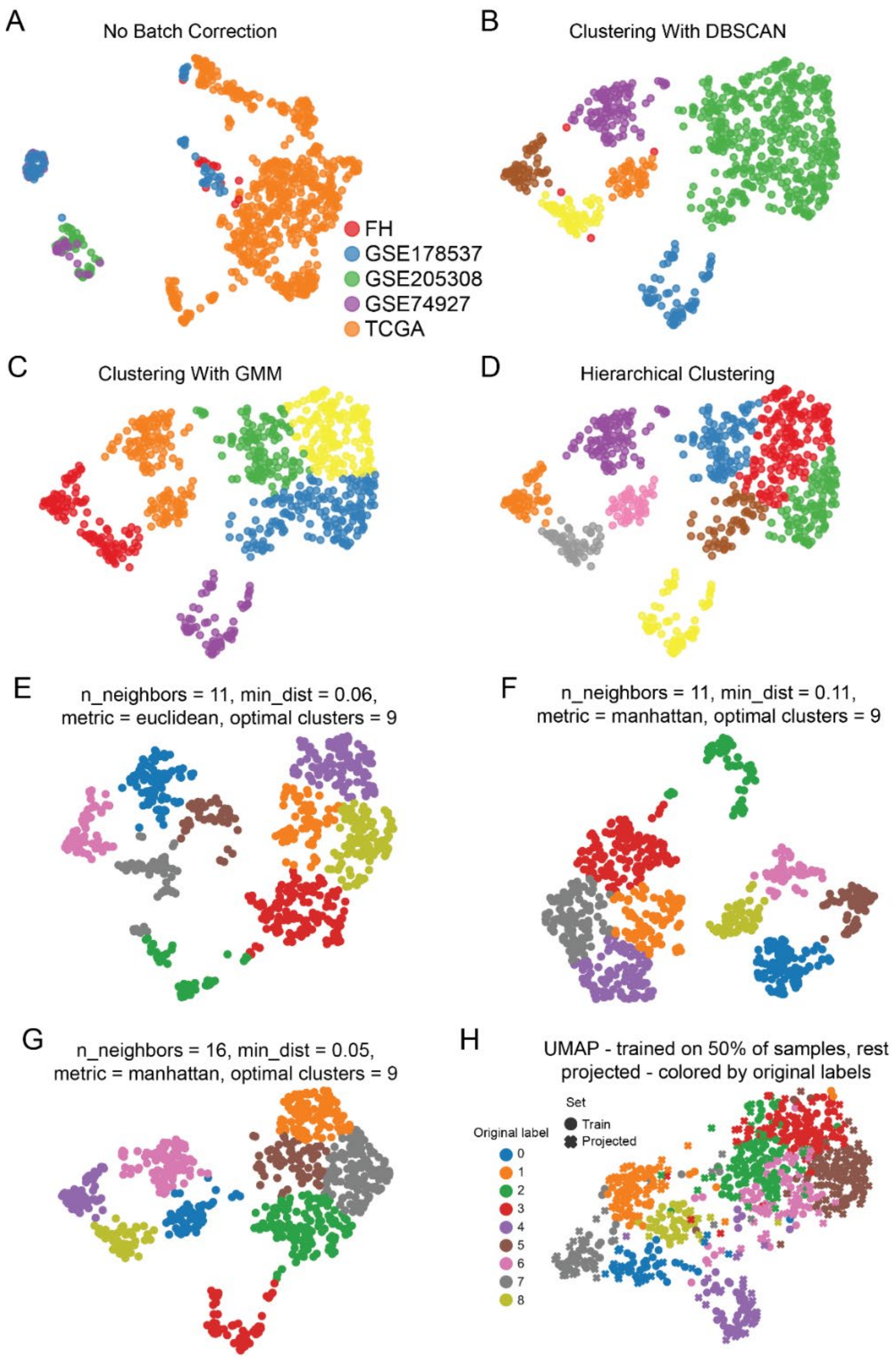

Fig. S1, related to Fig. 1. Comparative clustering and evaluation of cluster robustness in HNSCC. A UMAP of integrated HNSCC samples without batch correction, with colors denoting dataset of origin (FHCC, GEO datasets, TCGA). **B** UMAP of HNSCC samples clustered using DBSCAN, with colors indicating distinct clusters. **C** UMAP of HNSCC samples clustered using Gaussian Mixture Modeling (GMM), showing separation into distinct groups. **D** UMAP of HNSCC samples clustered using hierarchical clustering, with each color representing a cluster. **E-G** UMAPs generated using alternative UMAP parameter settings to evaluate the stability of cluster structure and the optimal number of clusters (k) using the silhouette method. **H** UMAP generated from a randomly sampled 50% subset of samples, with the remaining samples projected onto the embedding and colored according to their original cluster labels to assess clustering robustness.

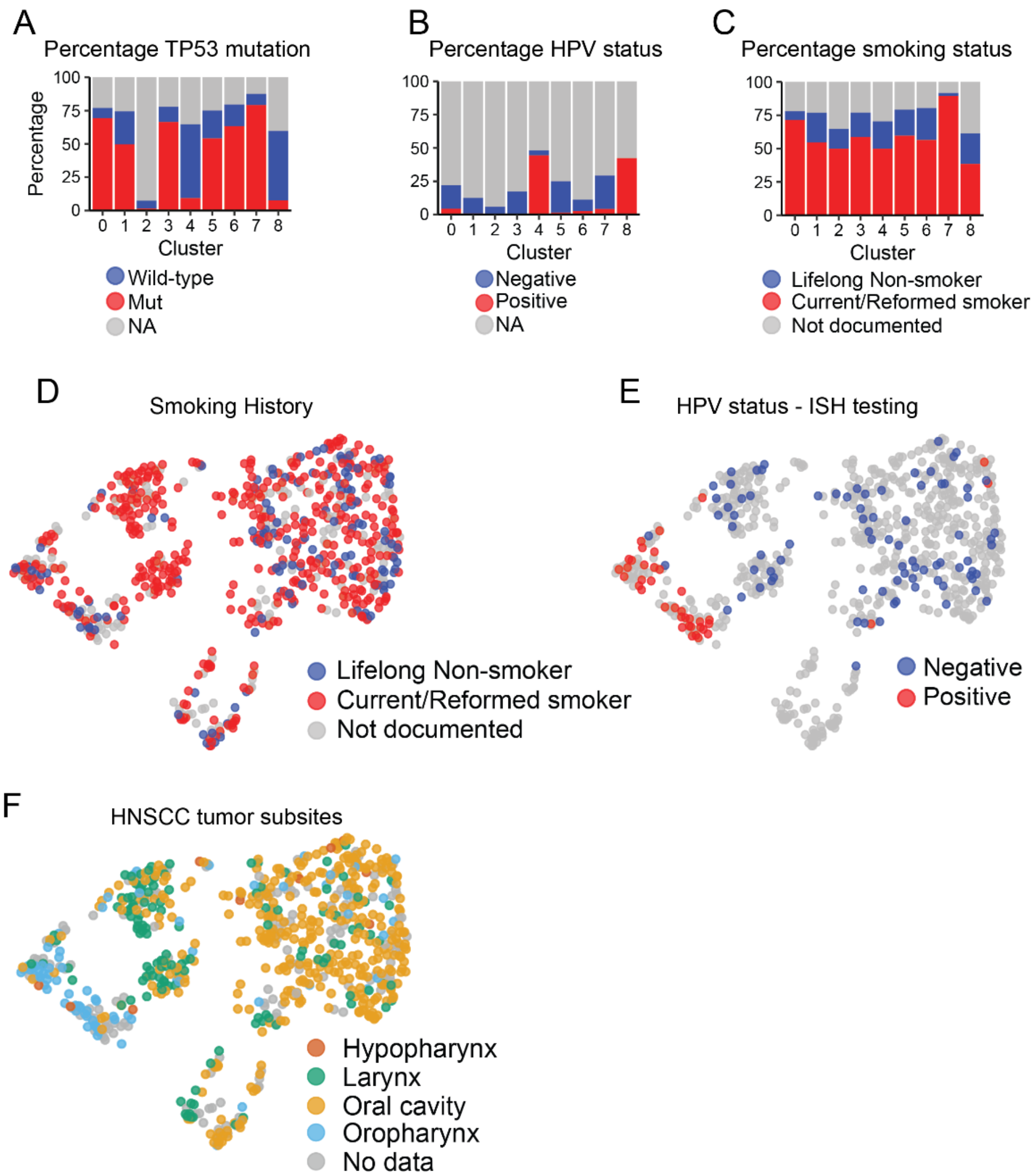

Fig. S2, related to Fig. 1. Clinical and molecular characteristics of HNSCC clusters. A Bar plots showing the percentage of TP53 mutation status (wild-type, mutant, NA) across clusters. **B** Bar plots showing the percentage of HPV status (positive, negative, NA) across clusters. **C** Bar plots showing the percentage of smoking status (lifelong non-smoker, current/reformed smoker, not documented) across clusters. **D** UMAP indicating smoking history, with samples colored by smoking status. **E** UMAP indicating HPV status from ISH testing, with samples colored by HPV-positive or -negative classification. **F** UMAP of HNSCC samples colored by tumor anatomical subsite (hypopharynx, larynx, oral cavity, oropharynx, or no data)

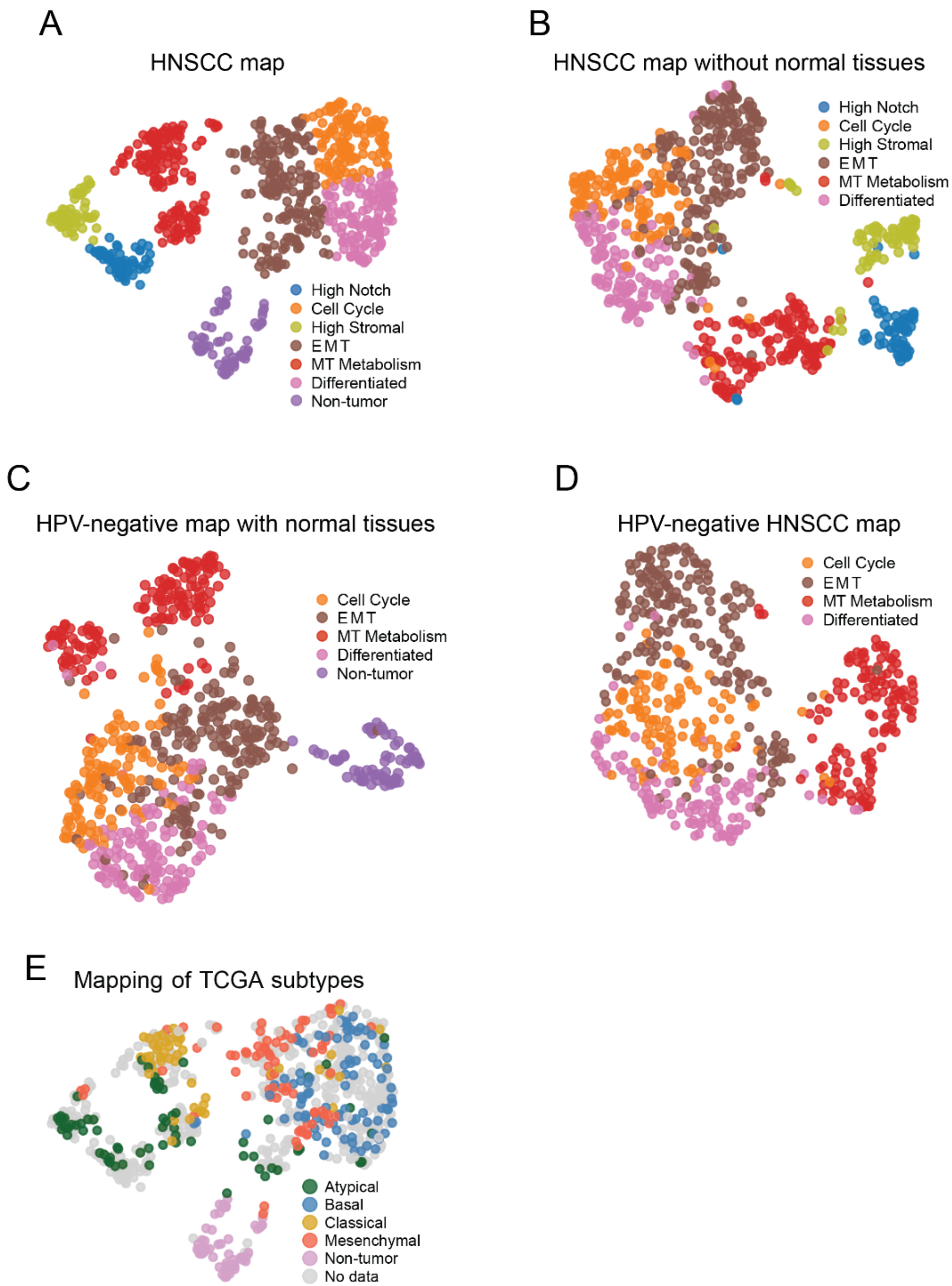

Fig. S3, related to Fig. 2. UMAP structure of integrated HNSCC cohorts and comparison with TCGA classifications. A UMAP of integrated HNSCC samples including normal tissues, colored by subtype. **B** UMAP of HNSCC samples after removal of normal tissues, showing the distribution of subtypes. **C** UMAP of HPV-negative samples including normal tissues. **D** UMAP of HPV-negative HNSCC tumor samples after removal of HPV-positive and normal tissues, showing the structure within HPV-negative tumors. **E** UMAP showing mapping of TCGA-HNSCC subtypes (Atypical, Basal, Classical, and Mesenchymal) onto the integrated HNSCC dataset.

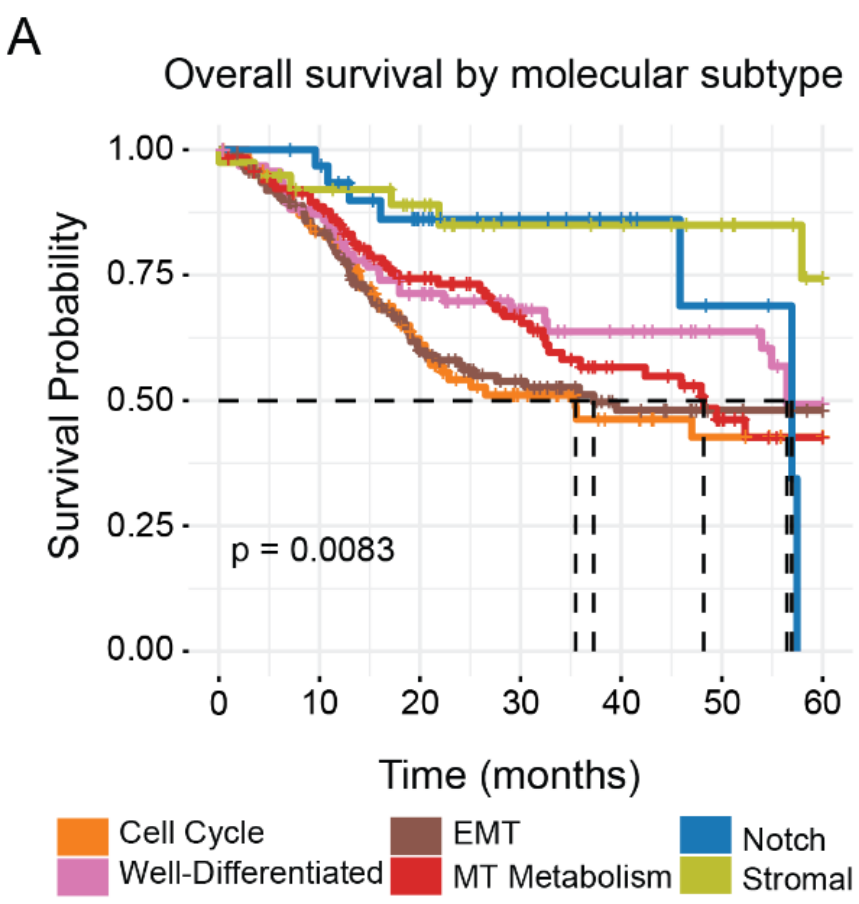

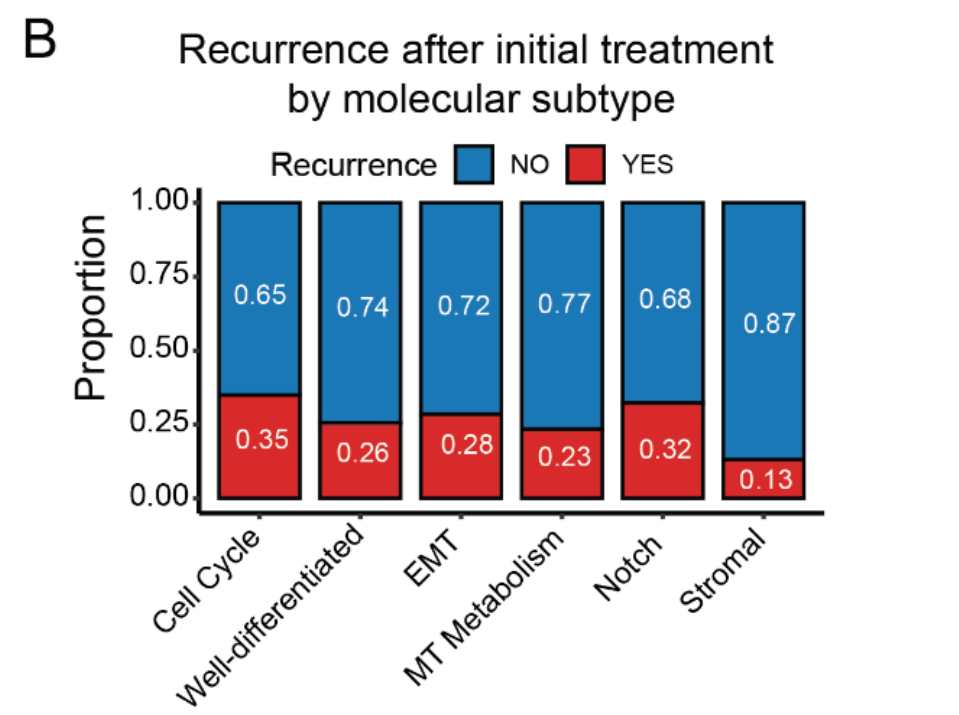

Distribution of Recurrence after initial treatment: chi-square p=0.179

	Cell Cycle	Well-Differentiated	EMT	MT Metabolism	Notch	Stromal
No recurrence	54	61	88	75	21	33
Recurrence after treatment	29	21	35	23	10	5

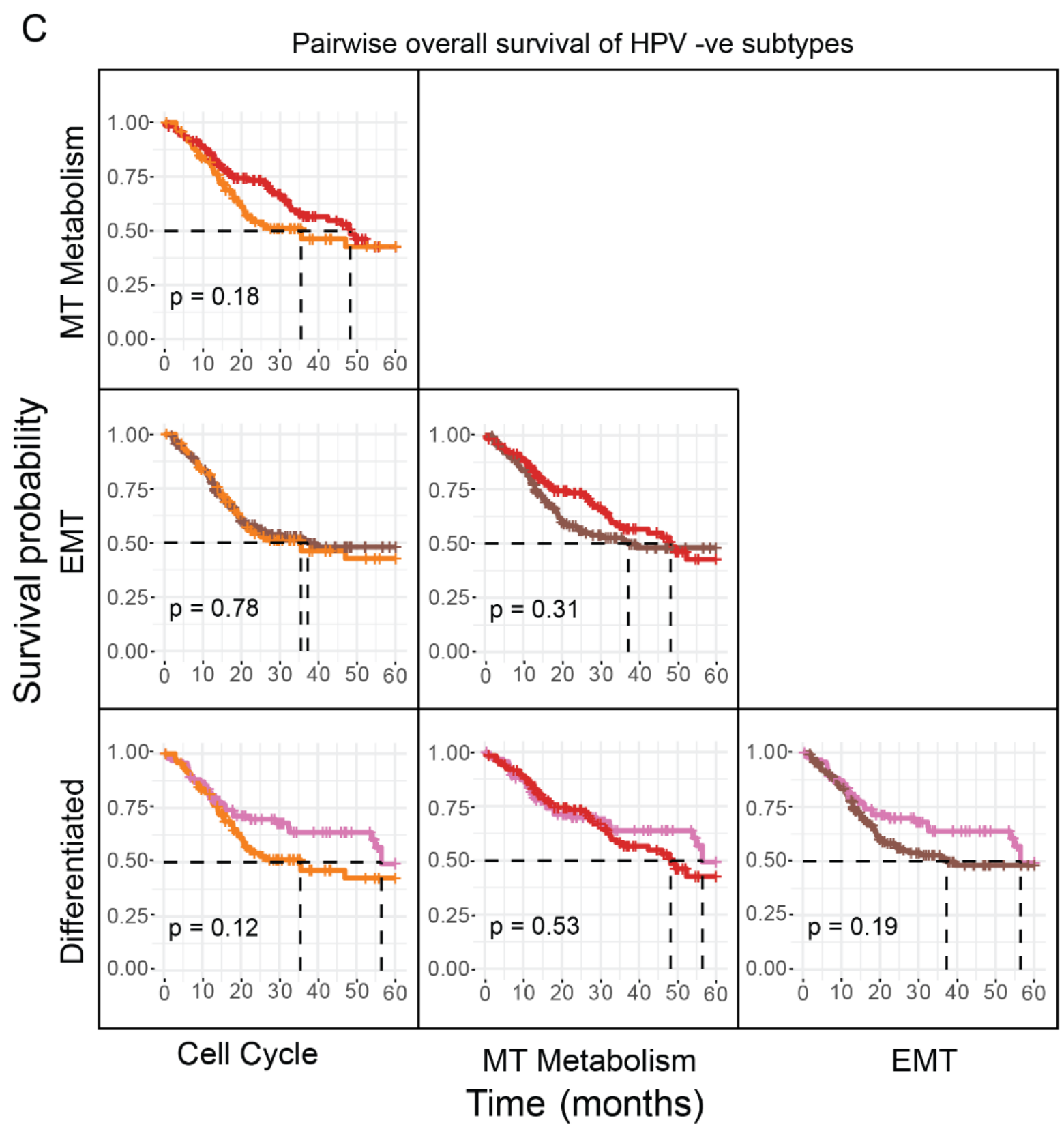

Fig. S4, related to Fig. 2. Clinical outcomes associated with HNSCC molecular subtypes A Kaplan-Meier overall survival curves for HNSCC patients stratified by molecular subtype (Cell Cycle, Well-Differentiated, EMT, MT Metabolism, Notch, and Stromal). P-value calculated using the log-rank test. **B** Proportion of patients with recurrence after initial treatment across molecular subtypes. Bars indicate the fraction of patients with or without recurrence. The contingency table below shows the distribution of recurrence events across subtypes (chi-square test).**C** Pairwise Kaplan-Meier comparisons of overall survival among HPV-negative molecular subtypes. P-values were calculated using the log-rank test.

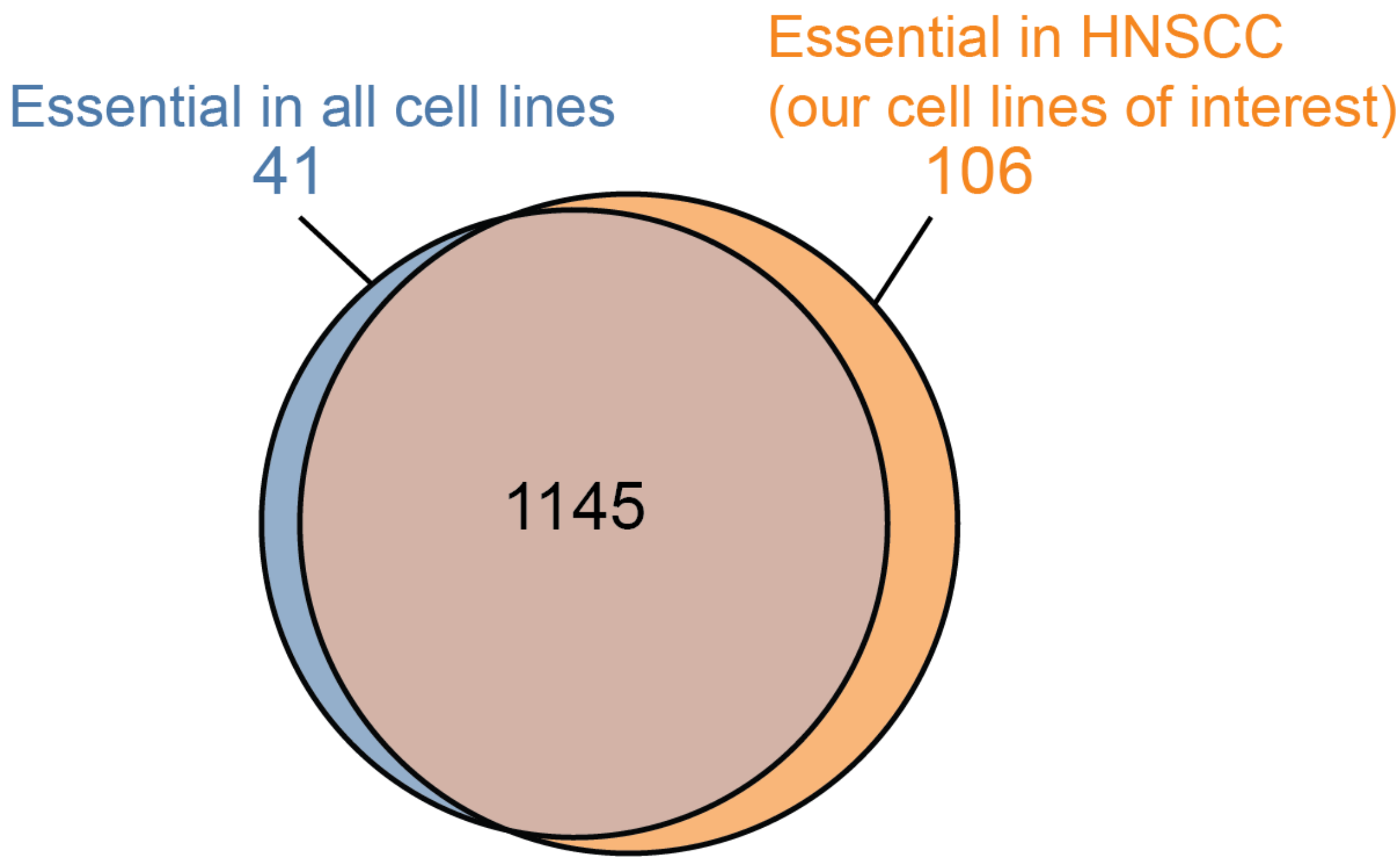

Fig. S5, related to Fig. 4. Overlap of CRISPR-defined essential genes in HNSCC vs. other cancers. Venn diagram showing genes essential in HNSCC (n=18) and all cell lines (n=1178). Essentiality is defined as present in ≥80% of all cell lines, matching the essentiality threshold used in the HNSCC subtype-specific analysis.

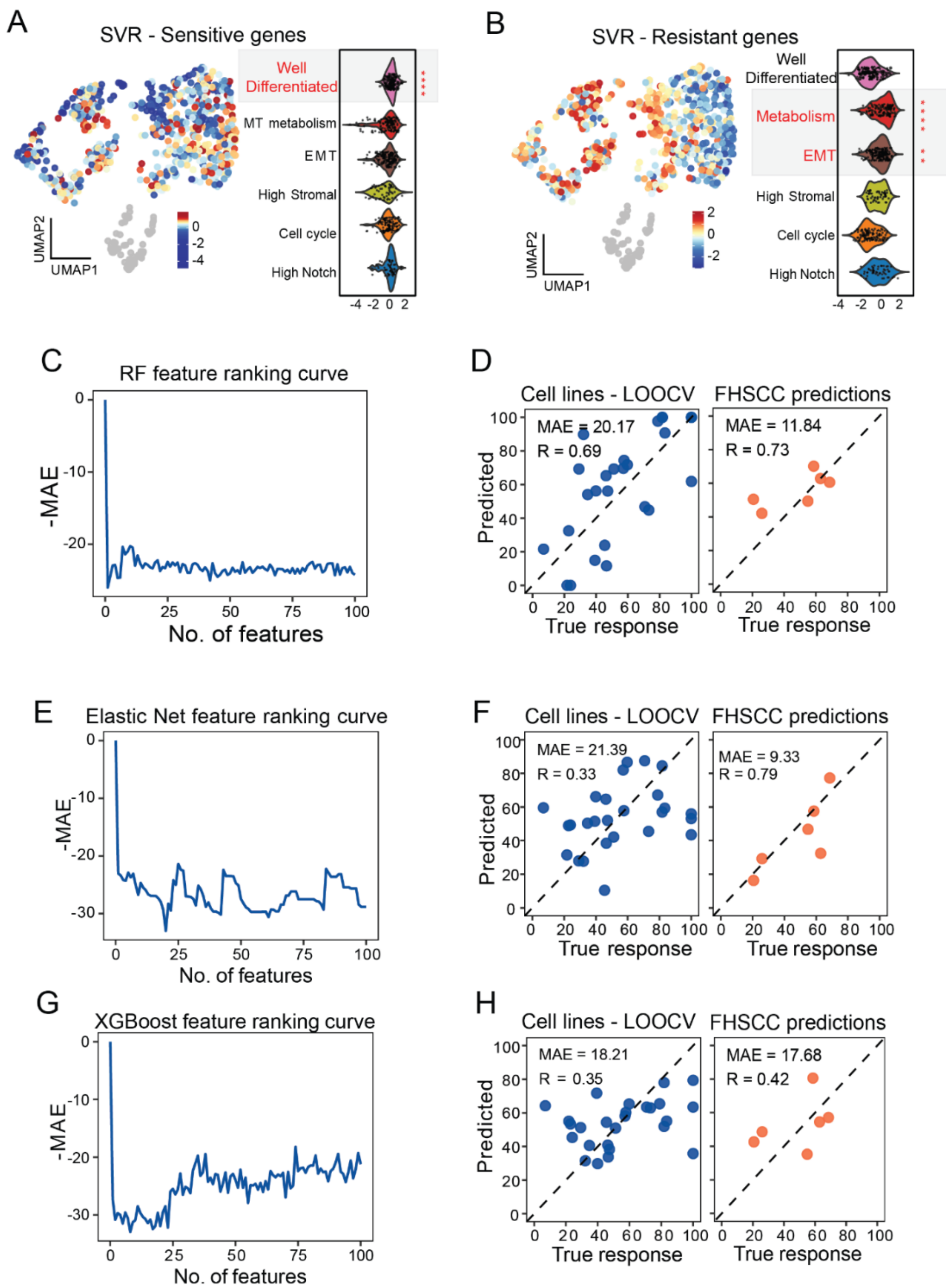

Fig. S6, related to Fig. 6. Comparative performance of machine learning models for predicting erlotinib sensitivity. A UMAP visualization and violin plots of ssGSEA z-scores for sensitivity-associated (positive coefficient) SVR genes, showing subtype-specific enrichment. **B** UMAP visualization and violin plots of ssGSEA z-scores for resistance-associated (negative coefficient) SVR genes, with significant enrichment across specific subtypes. **C** Feature ranking curve showing cross-validation MAE versus number of features for the Random Forest model. **D** Scatter plots of predicted versus true erlotinib responses for cell lines (left, LOOCV) and FHCC patient samples (right) using the Random Forest regression model. **E** Feature ranking curve showing cross-validation MAE versus number of features for the Elastic Net model. **F** Scatter plots of predicted versus true erlotinib responses for cell lines (left, LOOCV) and FHCC patient samples

(right) using the Elastic Net regression model. **G** Feature ranking curve showing cross-validation MAE versus number of features for the XGBoost model. **H** Scatter plots of predicted versus true erlotinib responses for cell lines (left, LOOCV) and FHCC patient samples (right) using the XGBoost regression model.
